# Supplementary material for: A neuronal mechanism controlling the choice between feeding and sexual behaviors in Drosophila
Source: Curr Biol. 2021 Oct 11;31(19):4231–4245.e4. doi: 10.1016/j.cub.2021.07.029 (PMC8538064; doi:10.1016/j.cub.2021.07.029)
Supplement: Document S1. Figures S1–S3 and Table S1 [file mmc1.pdf]

Current Biology, Volume *31*

Supplemental Information

A neuronal mechanism controlling the choice between feeding and sexual behaviors in *Drosophila*

Sherry J. Cheriyamkunnel, Saloni Rose, Pedro F. Jacob, Lauren A. Blackburn, Shaleen Glasgow, Jacob Moorse, Mike Winstanley, Patrick J. Moynihan, Scott Waddell, and Carolina Rezaval

Supplementary information

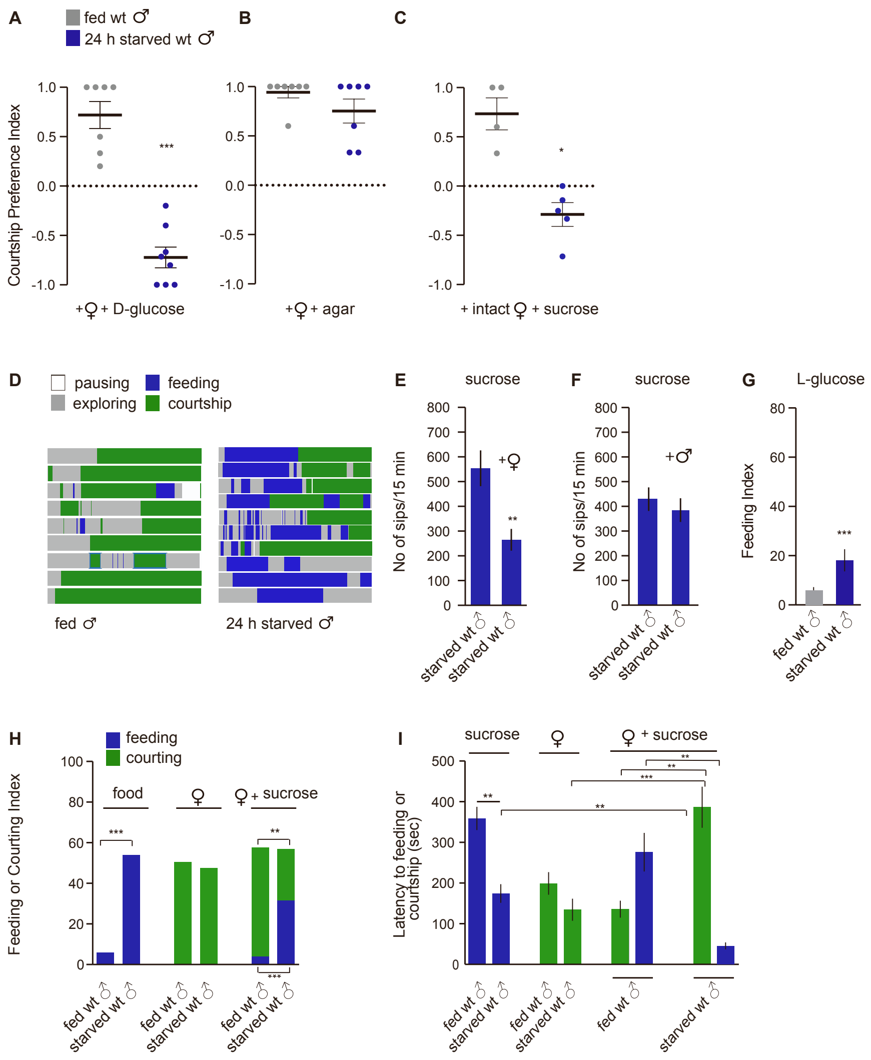

Figure S1. Analysis of feeding and courtship dynamics in sated and 24 h starved male flies, related to Figure 1.

(A-B) Courtship Preference Indices obtained from groups of fed and 24 h starved males choosing between courting a female or feeding on agar with D-glucose (A) (n=36-68 flies) or plain agar (B) (n=29-35 flies).

(C) Courtship Preference Indices obtained from groups of fed and 24 h starved males choosing between courting an intact, mobile female or feeding on sucrose (n=18-29).

(D) Representative ethograms of fed and starved Canton S males during 15 min in the presence of sucrose and a female (n=10).

(E) Quantitative feeding of flies on sucrose with or without a female, measured as number of sips in 15 min using flyPAD (16-22).

(F) Quantitative feeding of flies on sucrose with or without a decapitated male, measured as the number of sips in 15 min using flyPAD (n=44-58).

(G) Percentage of time spent by fed or 24 h starved CS males feeding on L-glucose (n=26-29).

(H) Percentage of time spent feeding on sucrose or courting the female during the observation period, in the presence of either stimulus or both (n=29-33).

(I) Latency in seconds (s) to the preferred action (feeding or courtship) of 24 h starved or fed males (n=26-30).

All bar graphs are shown as mean ± SEM, whereas the mean percentage time spent feeding or courting is shown in (H). Scatter plots show Courtship Preference Indices obtained from different groups of flies treated as independent biological replicates. Lines show the mean and error bars indicate SEM. *$p < 0.05$, **$p < 0.01$, ***$p<0.001$ calculated using Mann-Whitney U tests for in (A, B, C, E, F and G) and Kruskal Wallis test followed by Dunn's post-hoc multiple comparison test performed in (H, I). Absence of * denotes non-significant data.

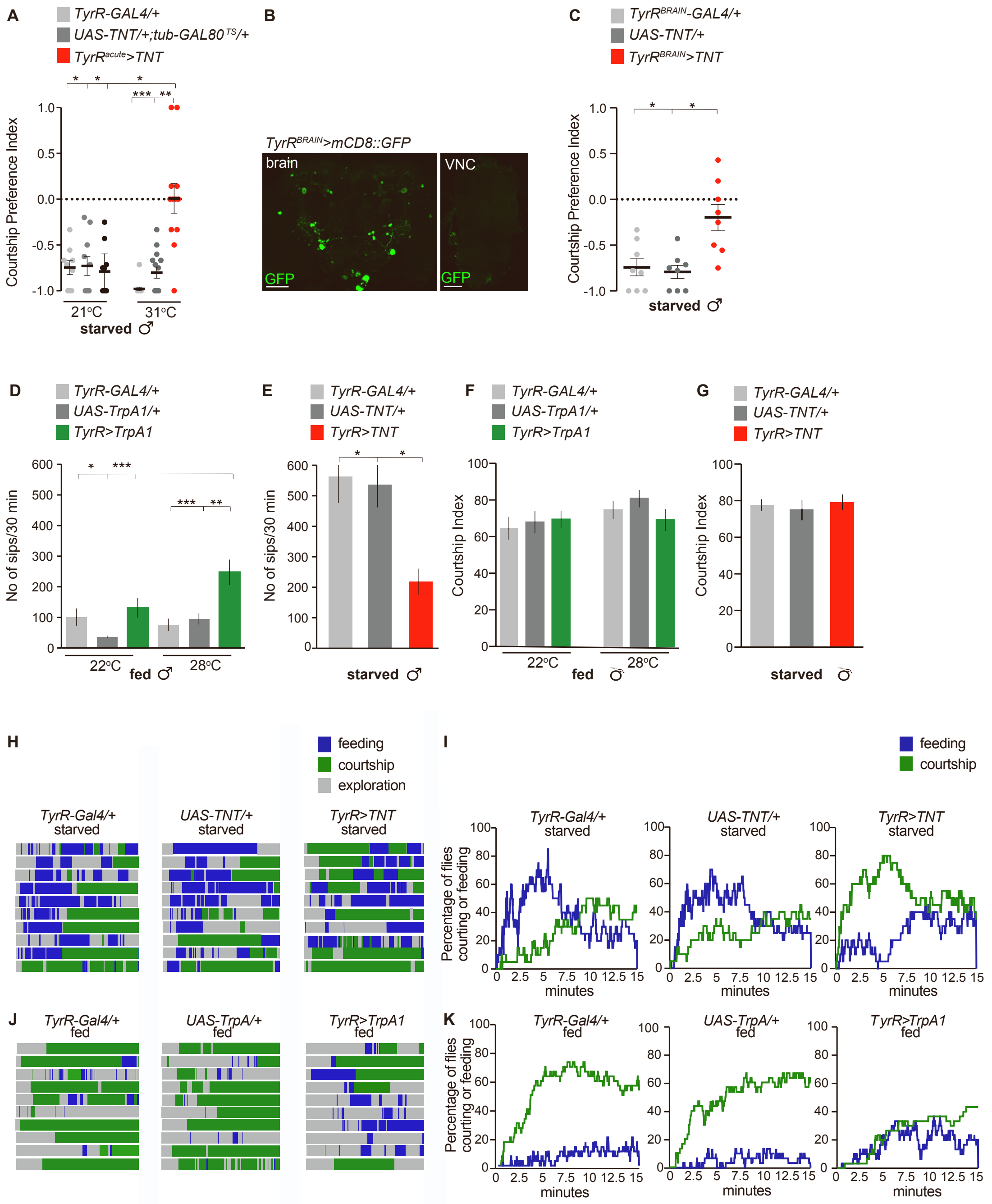

Figure S2. **Behavioral effects of manipulating TyrR and TyrR^{IPS} neurons in starved or fed male flies, related to Figure 2.**

(A) Acute silencing of TyrR neurons alters courtship preference. Courtship Preference Index of *UAS-TNT/+; tub-GAL80^{TS}/TyrR-GAL4* (*TyrR^{acute}>TNT*) males and genetic controls tested at 21°C and 31°C (n= 56-89).

(B) *UAS-mCD8-GFP* expressed under the control of *TyrR^{BRAIN}-GAL4* in the adult brain and ventral nerve cord. (Genotype used: *Otd-FLP/UAS>stop>mCD8::GFP;TyrR-GAL4/+).*

(C) Courtship Preference Index of *Otd-FLP/UAS>stop>TNT; TyrRGal4/+* (*TyrR^{brain}>TNT*) males and genetic controls (n = 52-60).

(D) Food intake of fed *TyrR-GAL4/UAS-TrpA1* and genetic control males paired with 100 mM sucrose measured as sips taken in 30 min in a flyPAD setting, tested at the control (22°C) and activation (28°C) temperatures, respectively (n=34-73).

(E) Food intake as number of sips taken during 30 min in 24 h starved *TyrR-GAL4/UAS-TNT* and genetic control males using flyPAD (n=26-40).

(F) Time spent courting in 15 min of fed *TyrR-GAL4/UAS-TrpA1* males and controls (n=16-30).

(G) Time spent courting in 15 min of starved *TyrR-GAL4/UAS-TNT* males and controls (n=23-31).

(H) Representative ethograms of *TyrR-GAL4/UAS-TNT* males and their respective genetic controls during 15 min in the presence of sucrose and a female (n=20).

(I) Percentage of *TyrR-GAL4/UAS-TNT* males (and their genetic controls) displaying either courtship or feeding behavior during 15 min in the presence of sucrose and a female in 1 s time bins (n=20).

(H) Representative ethograms of *TyrR-GAL4/UAS-TrpA* males and their respective genetic controls during 15 min in the presence of sucrose and a female (n=20).

(I) Percentage of *TyrR-GAL4/UAS-TrpA* males (and their genetic controls) displaying either courtship or feeding behavior during 15 min in the presence of sucrose and a female in 1 s time bins (n=20).

Scatter plots show Courtship Preference Indices obtained from different groups of flies treated as independent biological replicates. Lines show the mean and error bars indicate SEM. *p < 0.05, **p < 0.01, ***p<0.001 calculated using Kruskal Wallis followed by Dunn’s post-hoc multiple comparison test in A, C, D, E, F and G. In A, D and F the means obtained from the experimental groups were compared to both genetic controls tested at the control and activation temperatures and to data from the same genotype at the control temperature.

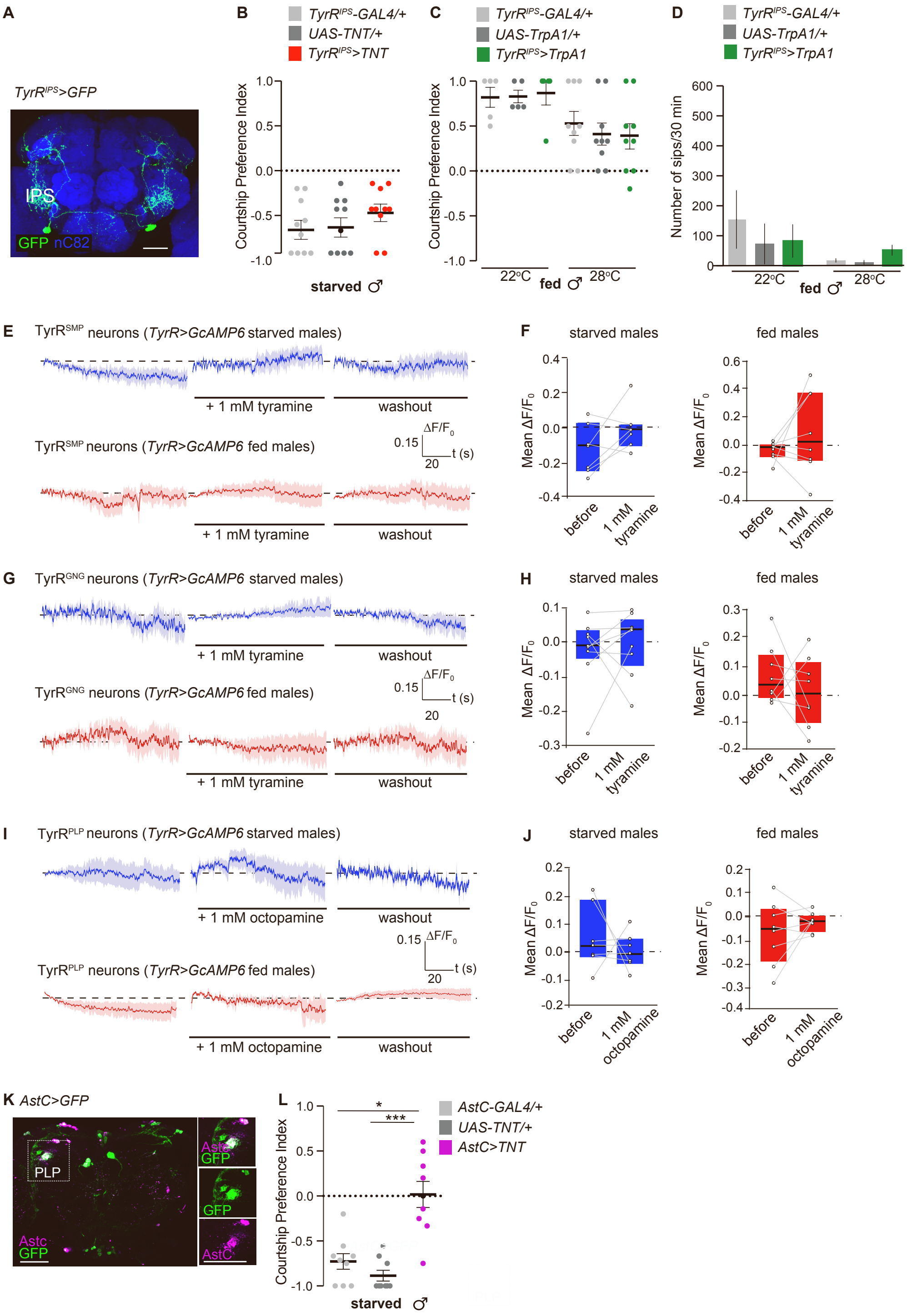

Figure S3. TyrR^{SMP}, TyrR^{GNG} and TyrR^{PLP} neurons response to tyramine and octopamine in starved and fed males, related to Figure 4.

(A) *UAS-mCD8-GFP* expressed under the control of IPS split-GAL4 in the adult brain. GFP staining (green); neuropil counterstained with anti-nC82 (blue). Scale bar= 50 μm.

(B) Courtship Preference Indices obtained from 24 h starved *IPS split-GAL4/UAS-TNT males*, choosing between feeding on sucrose and courting a female (n=57-64).

(C) Courtship Preference Indices of fed *IPS split-GAL4/UAS-TrpA1* and genetic control males, allowed to choose between feeding on sucrose and courting a female, at the control (22°C) and activation (28°C) temperatures (n= 40-60).

(D) Food intake of fed *IPS split-GAL4/UAS-TrpA1* and genetic control males paired with 100 mM sucrose measured as sips taken in 30 min in a flyPAD setting, at the control (22°C) and activation (28°C) temperatures (n=4-39).

(E) Application of 1 mM of tyramine (Tyr; middle panel) did not change the GCaMP6s Ca^{2+} response of $TyrR^{SMP}$ neurons in either starved (blue) or fed (red) males, when compared to the response before application (left panel). The $\Delta F/F_0$ represents the evoked fluorescence change from baseline. Traces were averaged from 8 flies. Solid line represents mean and shaded areas indicate SEM. Genotype tested: *UAS-GCaMP6s; TyrR-GAL4*.

(F) Quantification of the mean $\Delta F/F_0$ responses of $TyrR^{SMP}$ neurons using GCaMP6s over 90 s before (pre) and after the application of Tyr (1 mM Tyr) in starved (blue) and fed (red) males. No changes to 1 mM Tyr were observed in starved (n=8) or fed males (n=8).

(G) Application of 1 mM of tyramine (Tyr; middle panel) did not change the GCaMP6s Ca^{2+} response of $TyrR^{GNG}$ neurons in starved (blue) or fed (red) males, in comparison to the response before application (left panel). Traces were averaged from 8-9 flies. Genotype tested: *UAS-GCaMP6s; TyrR-GAL4*.

(H) Quantification of the mean $\Delta F/F_0$ responses of $TyrR^{GNG}$ neurons using GCaMP6s over 90 s before (pre) and after the application of Tyr (1 mM Tyr) in starved (blue) and fed (red) males. No changes to 1 mM Tyr were observed in starved (n=9) or fed males (n=8).

(I) Application of 1 mM of octopamine (middle panel) did not change the GCAMP6s Ca^{2+} response of $TyrR^{PLP}$ neurons in both starved (blue) and fed (red) males, in comparison to the response before application (left panel). Traces were averaged from 7-8 flies. Genotype tested: *UAS-GCaMP6s/61A01-LexA; LexAopGa80/TyrR-GAL4.*

(J) Quantification of the mean $\Delta F/F_0$ responses of $TyrR^{PLP}$ neurons (measured by GcAMP6s) over 90 s before (pre) and after the application of Oct (1 mM Oct) in starved (blue) and fed (red) males. No changes to 1 mM Oct were observed in starved (n=7) or fed males (n=8).

(K) UAS-mCD8-GFP expressed with AstC-GAL4 (III) in the adult brain. Anti-GFP (green) and anti-AstC (magenta) staining is shown. Scale bars= 50 μm or 25 um, insets.

(L) Courtship Preference Indices of 24 h starved *AstC-GAL4(III)/UAS-TNT* and genetic controls (n=50-61).

Lines show the mean and error bars indicate SEM. All boxplots represent 25th to 75th percentile and black line as the mean. *$p < 0.05$, **$p < 0.01$, ***$p<0.001$ calculated using paired t-test in (F, H and J for fed), Wilcoxon matched-pairs signed rank test in (J for starved) and Kruskal Wallis test followed by Dunn's post-hoc multiple comparison in (B, C, D and L). Absence of * denotes non-significant data.

Table S1: List of primers used for qRT-PCR in this study, Related to STAR Methods.

Primer	Sequence
Rp15 forward	AGG ATG CAC TTA TGG CAA GC
Rp15 reverse	GCG CAA TCC AAT ACG AGT TC
Tβh forward	ACA ATG TAC GTG GTT TGG GC
Tβh reverse	AGT ATC TTG TCG GCC CGT AG
Tdc2 forward	ACG CAT TGG CAG CAT CCT C
Tdc2 reverse	TGG CAG CAA GCA TCG TGA C
