# Supplementary material for: A neuronal mechanism controlling the choice between feeding and sexual behaviors in Drosophila
Source: Curr Biol. 2021 Oct 11;31(19):4231–4245.e4. doi: 10.1016/j.cub.2021.07.029 (PMC8538064; doi:10.1016/j.cub.2021.07.029)
Supplement: Document S2. Article plus supplemental information [file mmc4.pdf]

Current Biology

Article

A neuronal mechanism controlling the choice between feeding and sexual behaviors in *Drosophila*

Graphical abstract

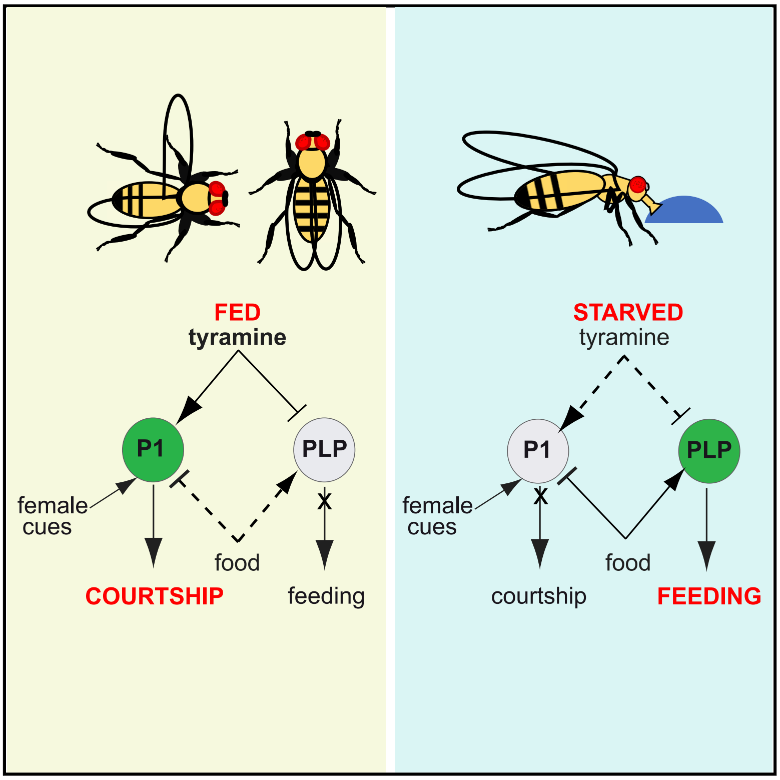

Highlights

- *Drosophila* males deprived of both food and sex have competing needs
- Choosing between feeding or mating is modulated by food quality and internal drive
- Tyramine signaling mediates the choice between feeding and courtship
- Antagonism between feeding and courtship neurons determines the behavior selected

Authors

Sherry J. Cheriyamkunnel, Saloni Rose, Pedro F. Jacob, ..., Patrick J. Moynihan, Scott Waddell, Carolina Rezaval

Correspondence

c.rezaval@bham.ac.uk

In brief

How are alternative options evaluated in the brain and specific actions prioritized? Cheriyamkunnel et al. describe a novel neural mechanism by which fruit flies balance and prioritize the competing needs of feeding and mating by integrating nutrient availability and motivational drives.

Cheriyamkunnel et al., 2021, Current Biology *31*, 4231–4245
October 11, 2021 © 2021 The Authors. Published by Elsevier Inc.
https://doi.org/10.1016/j.cub.2021.07.029

CellPress

Current Biology

CellPress
OPEN ACCESS

Article

A neuronal mechanism controlling the choice between feeding and sexual behaviors in *Drosophila*

Sherry J. Cheriyamkunnel,[1,3] Saloni Rose,[1,3] Pedro F. Jacob,[2,3] Lauren A. Blackburn,[1] Shaleen Glasgow,[1] Jacob Moorse,[1] Mike Winstanley,[1] Patrick J. Moynihan,[1] Scott Waddell,[2] and Carolina Rezaval[1,4,5,*]

[1]School of Biosciences, University of Birmingham, Birmingham B15 2TT, UK
[2]Centre for Neural Circuits and Behaviour, University of Oxford, Oxford OX1 3SR, UK
[3]These authors contributed equally
[4]Twitter: @crezaval
[5]Lead contact
*Correspondence: c.rezaval@bham.ac.uk
https://doi.org/10.1016/j.cub.2021.07.029

SUMMARY

Animals must express the appropriate behavior that meets their most pressing physiological needs and their environmental context. However, it is currently unclear how alternative behavioral options are evaluated and appropriate actions are prioritized. Here, we describe how fruit flies choose between feeding and courtship; two behaviors necessary for survival and reproduction. We show that sex- and food-deprived male flies prioritize feeding over courtship initiation, and manipulation of food quality or the animal's internal state fine-tunes this decision. We identify the tyramine signaling pathway as an essential mediator of this decision. Tyramine biosynthesis is regulated by the fly's nutritional state and acts as a satiety signal, favoring courtship over feeding. Tyramine inhibits a subset of feeding-promoting tyramine receptor (TyrR)-expressing neurons and activates P1 neurons, a known command center for courtship. Conversely, the perception of a nutritious food source activates TyrR neurons and inhibits P1 neurons. Therefore, TyrR and P1 neurons are oppositely modulated by starvation, via tyramine levels, and food availability. We propose that antagonistic co-regulation of neurons controlling alternative actions is key to prioritizing competing drives in a context- dependent manner.

INTRODUCTION

Animals must make crucial behavioral choices on a minute-by-minute basis to survive in a changing environment. Many of these decisions can be binary.[1] For example, on perceiving a food source, an organism can choose to avoid or initiate feeding. However, animals are often confronted with environmental cues and internal drives that stimulate incompatible behaviors (e.g., feeding versus mating or threat avoidance).[2] It is therefore important to understand how alternative options are evaluated in the brain and how specific actions are prioritized.

Studies in invertebrates suggest that behavioral selection can be achieved by reciprocal inhibition between neurons controlling incompatible behaviors.[1,3] For instance, in the predatory sea slug *Pleurobranchaea*, neurons of the feeding network suppress withdrawal command neurons, promoting feeding over escape responses.[4] Although inhibition between response-dedicated neurons may exist to establish a "behavioral hierarchy" that prioritizes crucial actions over others, evidence suggests that "multifunctional" neurons in distributed networks can also mediate behavioral decisions.[1,3] For example, in the leech, swimming and crawling are mutually exclusive behaviors that are partly controlled by overlapping central pattern generator interneurons, which can be reconfigured to elicit either behavior.[3] However, whether these principles apply to the brains of other organisms remains to be explored.

Several studies have identified neurons or mechanisms mediating the choice between competing behaviors in *Drosophila*.[5–12] For instance, coordination between walking and feeding behaviors, which cannot be simultaneously expressed, depends on a single pair of interneurons in the ventral nerve cord.[5] Activating these neurons suppresses feeding while the fly is walking, whereas inhibition induces feeding at the expense of locomotion. Other studies have investigated antagonism between sleep and feeding.[7,10–12] Food-deprived flies forgo sleep in favor of feeding, whereas they are likely to sleep after a meal.[12] Activation of allatostatin-A expressing cells promotes sleep and inhibits feeding.[7] Last, a recent study explored how flies prioritize the need to seek water or food when they are thirsty and/or hungry.[9] Expressing the relevant resource-seeking memory involves deprivation-state specific neuropeptidergic modulation of different subsets of dopaminergic neurons.

An important behavioral conflict also arises when an animal is deprived of both food and sex and therefore must decide whether to prioritize searching for resources to feed or mate. Although an energy deficit often represents an immediate threat for survival, it is unclear if reproduction can override

© 2021 The Authors. Published by Elsevier Inc.
This is an open access article under the CC BY license (http://creativecommons.org/licenses/by/4.0/).

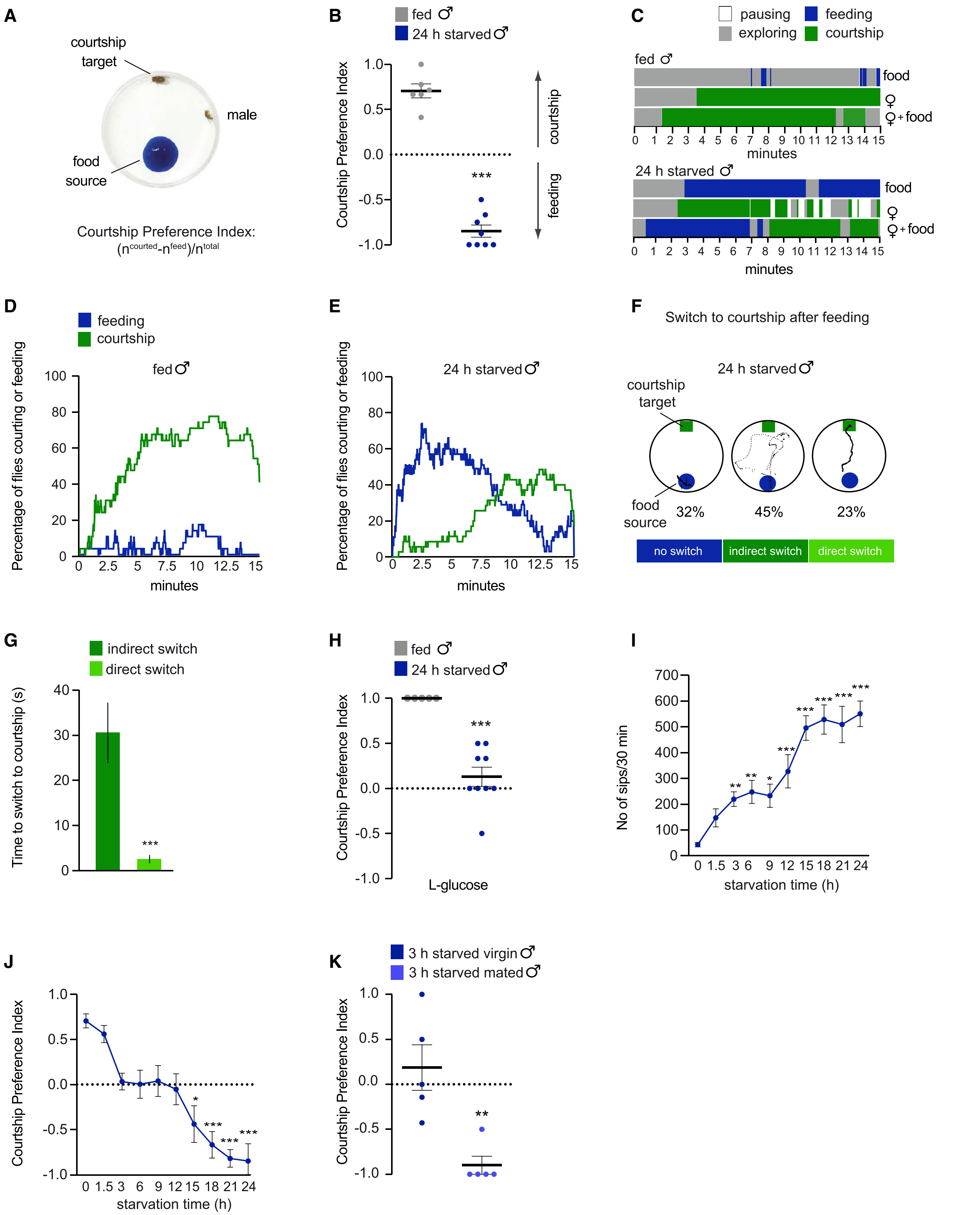

Figure 1. Male flies deprived of food and females prioritize feeding over courtship in a context-dependent manner

(A) In our behavioral choice assay, 24-h starved or fed wild-type male flies are allowed to choose between feeding from a 100 mM sucrose-containing food source and courting a decapitated virgin female (courtship target).

(legend continued on next page)

CellPress
OPEN ACCESS

hunger-directed behaviors under certain contexts. Prior studies have explored how internal state influences female receptivity[13–17] and feeding preference and exploration.[18–20] Yet how food deprivation affects the choice between feeding and mating in *Drosophila* remains unexplored.

Male fruit flies engage in an elaborate innate courtship ritual through which they evaluate the suitability of a potential mate by exchanging a variety of sensory cues.[21] Male flies are highly motivated to court, even toward suboptimal targets,[22–25] and find sex rewarding.[26] Neurons expressing the sex determination genes *doublesex* and *fruitless* are known to receive, process, and transfer information that controls male courtship.[21] A subgroup of ~20 male-specific P1 neurons serves as a command center that initiates courtship in response to external sensory cues, such as pheromones from conspecific mates and internal states including their mating status.[27–34]

Drosophila feeding behavior is comprised of a series of food seeking and consumption subprograms.[35] Detection of food and subsequent feeding is initiated by activation of gustatory neurons on the fly's legs and proboscis. Neuromodulatory systems like dopamine, neuropeptide F (dNPF), and insulin-like peptides (DILPs) play an integral role in fine-tuning the feeding program in response to the nutritional state of the fly. Satiety signals terminate feeding and disengagement from the food source.[35,36] However, it remains unclear how the fly chooses between feeding and courting.

Here, we established an assay to examine the neuronal mechanisms that underlie *Drosophila*'s choice between courtship and feeding. We show that sex- and food-deprived male flies prioritize initiating feeding over courtship, and this behavioral decision can be reshaped by internal needs and current environmental context. We discover tyramine signaling to be an important mediator of the feeding or courtship choice. Feeding-promoting tyramine receptor (TyrR) neurons and courtship-promoting P1 neurons are antagonistically modulated by tyramine and food detection, which matches behavior with starvation state and resource availability.

RESULTS

Feeding overrides courtship in a novel behavioral choice assay in *Drosophila*

To investigate the neural mechanisms controlling choice between competing options, we developed an assay in which a wild-type (Canton-S) *Drosophila* male can either court or feed (Figure 1A). Individual male flies were socially isolated post-eclosion and sex- and food-deprived in vials containing a water source for 24 h prior to experimentation. Males were then given the choice to feed on 100 mM sucrose or court a beheaded virgin female. Headless females are attractive courtship targets[23] but are mostly immobile and thus maximize the observation of male behavior. Sex- and 24-h food-deprived males showed a clear preference for feeding over courtship initiation, whereas most of their fed counterparts chose to court the female (courtship preference index [CPI] −0.8 versus +0.7) (Figures 1A and 1B; Videos S1 and S2). Similar results were obtained when starved males were presented with another nutritive sugar (D-glucose) (CPI −0.7 versus +0.7) (Figure S1A), demonstrating that sex- and food-deprived flies preferentially eat nutritious food rather than court a female. Feeding preference was not due to dehydration during starvation, because most 24-h starved males exposed to a female and plain agar engaged in courtship (CPI ~+0.8 versus +0.9) (Figure S1B). Female behavior did not significantly influence the male's choice as starved males allowed to choose between sucrose and courting an intact mobile female still prioritized feeding over courtship initiation compared to fed flies (CPI −0.3 versus +0.6) (Figure S1C).

Starved males switch to courtship after feeding

We next tested whether starved flies that initially fed would switch to courtship after feeding for a certain period and/or whether they would feed to satiation before attending to the female. To assess the males' behavioral dynamics over time, we created ethograms of the behavior of individual fed and 24-h starved males exposed to a female and food during a 15-min

(B) Courtship Preference Indices obtained from groups of 24-h starved and fed males, allowed to choose between courting a female and eating sucrose. Each dot in the scatterplot represents the CPI obtained from a group of flies (n = 52–56 total flies per condition).
(C) Representative ethograms illustrating the temporal dynamics of a fly feeding or courting during 15 min, either in the presence of sucrose, a female, or both resources.
(D and E) Temporal dynamics of fed (D) and 24-h starved (E) flies in the presence of sucrose and a female. Percentage of males performing either courtship or feeding over a time period of 15 min in 1-s time bins (n = 30–35).
(F) Representative spatial maps depicting the trajectory of a 24-h starved male during its switch to courtship after food ingestion. Percentages indicate the proportion of males that only fed during the observation period ("no switch"), males that switched to courtship after a short period of exploration ("indirect switch") and males that immediately started to court as they left the food ("direct switch") (n = 31).
(G) Indirect and direct switch to courtship depicted as time taken in seconds (s) to switch to courtship after ingestion finished (n = 31).
(H) Courtship preference indices obtained from groups of 24-h starved or fed flies choosing between feeding on L-glucose and courting a female (n = 14–35).
(I) Quantitative analysis of feeding of flies on sucrose after different intervals of starvation. Food intake was measured as the number of sips over 30 min using flyPAD. Duration of starvation is displayed on the horizontal axis (n = 14–44).
(J) Courtship preference indices of different groups of progressively starved flies allowed to choose between eating sucrose and courting a female (n = 26–56).
(K) Courtship preference indices obtained from groups of 3 h starved virgin or recently mated males choosing between ingesting sucrose and courting a decapitated female (n = 18–27).
Scatterplots show courtship preference indices obtained from different groups of flies treated as independent biological replicates. Lines show the mean and error bars indicate SEM. *p < 0.05, **p < 0.01, ***p < 0.001 calculated using Mann-Whitney U tests for data in (B), (G), (H), and (K) and Kruskal-Wallis test followed by Dunn's post hoc multiple comparison test in (I) and (J), where group medians were compared to the median of fed control groups. Absence of asterisk (*) denotes non-significant data.
See also Figure S1 and Videos S1 and S2.

observation time. Fed males prioritized courtship throughout the observation time and occasionally fed in small bouts (Figures 1C, 1D, and S1D). Starved males initially fed for ~4.5 min on average then switched to courtship and continued to court the female for ~4 min (Figures 1C and S1D). This behavioral switch was robust and generalizable across the population of starved flies (Figure 1E).

We noted that starved males distributed their time equally between feeding and courtship (time spent feeding or courtship: 29% and 25%, respectively) (Figures 1C, 1E, and S1D). However, starved males fed for a shorter period when a female was present than when there was no female (Figures 1C and S1H). We therefore investigated whether the males feed more rapidly and efficiently when there is also a female nearby. For these analyses, we used fly proboscis and activity detector (flyPAD),[37] an automated method for quantifying food intake over time. We found that starved males simultaneously presented with a female and food reduce their food intake (number of sips), compared to baseline feeding levels in the absence of a mate (Figure S1E). This effect appears to be specific to the presence of a female and not food competition, because starved males did not significantly decrease their food intake in the presence of another male (Figure S1F). These results suggest that starved males do not feed more quickly and efficiently in the presence of a female. However, starved males initiated feeding ~4 times faster if a female was presented with food (Figures 1C and S1I). Finally, we noted that in the absence of food, starved males courted females to the same extent as fed males, and courtship was only reduced when males were presented with nutritious food (Figures 1C and S1H). This suggests that both starvation and food cues are required for males to strongly suppress courtship.

To further investigate how starved males prioritize their needs over time, we created spatial maps depicting the trajectory of their movement as they finished feeding (Figure 1F). Although ~32% of 24-h starved males only fed and did not switch to courtship during the observatory period (“no switch”), 45% engaged in courtship following a short period of exploration (“indirect switch”). Strikingly, 23% of starved males immediately started to court on leaving the food, taking a direct path to the female (“direct switch”) (Figures 1F and 1G). These findings indicate that most starved male flies distribute their time equally between courtship and feeding and quickly switch their attention between the resources when they are both available.

The quality of food, the fly’s nutritional state, and sex drive modulate behavioral choice

We established that sex- and 24-h food-deprived males initially engage in feeding as opposed to courtship. We next asked if this behavioral choice is influenced by external conditions (e.g., the nutritional value of the food) and motivational states (hunger and sex drive). To monitor the effect of food quality on this behavioral choice, we presented males with a female and L-glucose, a sugar that flies ingest (Figure S1G) but cannot metabolize.[38] Notably, starved males showed higher courtship preference when confronted with the low-calorie sugar (CPI −0.9 versus +0.1) (Figure 1H).

We next investigated whether the male’s choice to initiate feeding or courtship is modulated by the degree of starvation. We first used flyPAD to monitor how starvation affects feeding in solitary males. Males that were progressively starved from 1.5 to 24 h showed a gradual and significant increase in feeding (Figure 1I). In the presence of food and a potential mate, courtship preference decreased with starvation (slope = −0.061114, $p < 0.0001$, $R^2 = 0.6639$) (Figure 1J). A significant shift from courtship to feeding occurred after 15 h of starvation, as compared to non-starved males (CPI ~ −0.4).

Repeated copulations decrease a male fly’s drive to mate.[34] We therefore tested whether courtship motivation affects the initial choice between feeding and courtship. We chose a 3-h starvation interval where no clear preference for feeding or courtship is observed (Figure 1J) and evaluated the behavior of virgin and recently mated males (allowed to copulate once, 1 h prior to experimentation). Although no clear behavioral preference was evident in unmated males, mated males favored feeding over courtship (CPI +0.2 versus −0.9) (Figure 1K).

TyrR neurons promote feeding over courtship initiation

Our findings indicate that choice between courtship and feeding initiation can be fine-tuned by manipulating the fly’s nutritional state, recent sexual activity, and the quality of the available food source. We therefore reasoned that there must be a neural mechanism that integrates starvation satiation signals, food, and mating cues to modulate the behavioral decision between feeding and courtship.

To genetically manipulate the activity or synaptic output of candidate neurons, we used the GAL4/UAS system[39] to express the tetanus toxin light chain (TNT), which blocks evoked synaptic neurotransmission.[40] We initially performed a screen targeting neurons expressing neuromodulators, neuropeptides, or their receptors. Silencing neurons that express the receptor for the biogenic amine tyramine (TyrR neurons) resulted in a significantly larger proportion of 24-h starved *TyrR>TNT* males choosing to court the female instead of feeding, as compared to 24-h starved controls (CPI −0.01 versus −0.9 and −0.6) (Figure 2A). In contrast, inactivating TyrR neurons did not significantly affect the behavior of fed males (Figure 2B). To rule out defects caused by inhibiting these neurons throughout development, we restricted silencing of TyrR neurons to adult flies by combining TNT with the temperature-sensitive GAL80 ($GAL80^{TS}$) inhibitor of GAL4, expressed ubiquitously from a tubulin promoter.[41] *TyrR>TNT*;tub-$GAL80^{TS}$ flies and controls kept at 20°C did not show any differences in behavior (CPI −0.8 versus genetic controls −0.8 and −0.7) (Figure S2A). In contrast, adult *TyrR>TNT* males carrying tub-$GAL80^{TS}$ that were kept at 31°C and starved for 24 h showed increased courtship preference compared to controls (CPI ~0 versus genetic controls ~−1 and −0.8) (Figure S2A), recapitulating our findings with chronic TNT-mediated inhibition of TyrR neurons and thus ruling out any developmental etiology.

We next tested whether enforced activation of TyrR neurons induced the opposite behavioral effect to that observed with neuronal silencing. We expressed a UAS-transgene for the thermosensitive Ca^{2+} channel dTrpA[42] in TyrR neurons and evaluated the behavioral consequence of transient neuronal activation in courtship or feeding initiation. At the control temperature of 22°C, fed *TyrR>TrpA1* males behaved indistinguishably from controls (Figure 2D). However, at 28°C activation of TyrR neurons promoted feeding over courtship initiation in fed males

CellPress
OPEN ACCESS

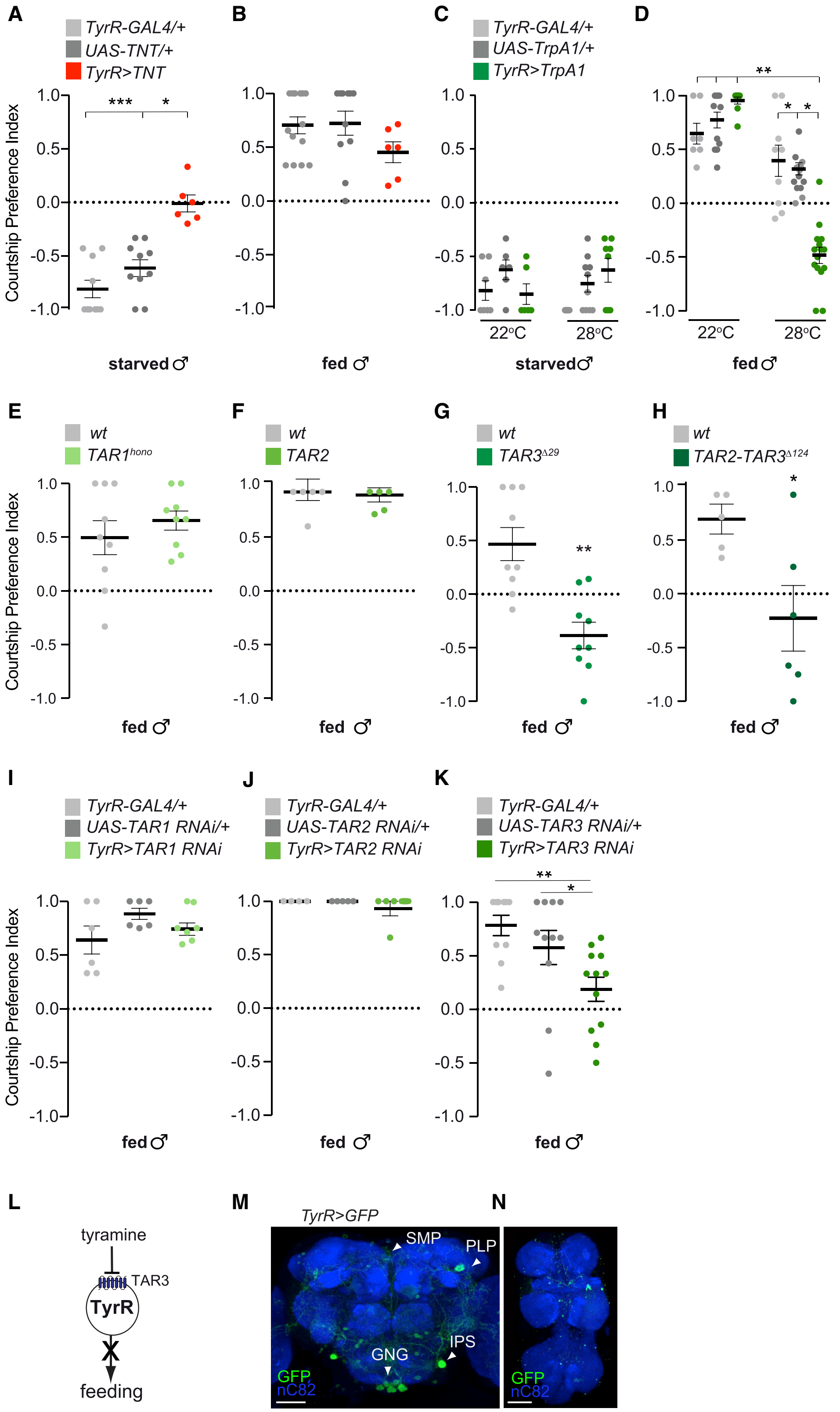

Figure 2. Artificial inhibition and activation of TyrR neurons alters behavioral choice in starved and sated male flies

(A and B) Courtship preference indices obtained from 24-h starved (A) or fed (B) *TyrR-GAL4>UAS-TNT* males, choosing between feeding on sucrose and courting a female (n = 65–106 in A and 53–93 in B).

(legend continued on next page)

(CPI −0.5 at 28°C versus +0.9 at 22°C) (Figure 2D). Artificial activation of TyrR neurons did not significantly affect the behavior of starved males (Figure 2C). Our studies demonstrate that activation of TyrR neurons promotes feeding over courtship in fed (but not starved) flies and TyrR inhibition promotes courtship over feeding in starved (but not fed) flies. Next, we tested whether manipulating TyrR neurons affects overall feeding levels. In the absence of a female, TyrR neuron activation increased feeding in fed males, whereas their inhibition decreased feeding in starved males (Figures S2D and S2E). However, in the absence of food, neither activating nor silencing TyrR neurons notably affected courtship levels (Figures S2F and S2G). These results argue that TyrR neurons promote feeding in starved males, and favor feeding over courtship initiation in the choice context.

Next, we tested whether the dynamics and distribution of the courtship and feeding behaviors change over 15 min upon inhibiting TyrR neurons in 24-h starved flies. As expected, starved control flies prioritized feeding over courtship for approximately half of the experiment before directing their preference to courtship (Figures S2H and S2I). In contrast, most starved *TyrR>TNT* flies favored courtship for ∼7.5 min, and then switched to feeding (Figures S2H and S2I). Therefore, silencing TyrR neurons does not completely abolish feeding but rather tips the initial behavioral choice toward courtship. On the other hand, activation of TyrR neurons in *TyrR>TrpA1* flies significantly reduced courtship initiation in the choice context (Figures S2J and S2K). These findings further show that activation of TyrR neurons can alter courtship without profoundly changing overall feeding.

TyrR neurons in the brain mediate the behavioral choice

The TyrR neural cluster is composed of ∼50 cells in the brain and a few neurons in the ventral nerve cord (VNC) (Figures 2M and 2N).[43] To assess the role of brain TyrR neurons in the behavioral choice, we combined TyrR-GAL4 and a brain-specifically expressed FLP recombinase (Otd-FLP)[44,45] with a UAS > stop > effector to restrict transgene expression to the brain. $TyrR^{BRAIN}$*>mGFP* intersected males showed the typical TyrR brain expression pattern but no labeling in the VNC (Figure S2B). Inhibiting the brain specific neurons in $TyrR^{BRAIN}$*>TNT* males decreased the courtship preference index (CPI ∼−0.2 versus genetic controls −0.7 and −0.8) (Figure S2C), supporting a role for brain TyrR neurons in the feeding-courtship behavioral selection.

TAR3 receptor is required in TyrR neurons to prioritize feeding over courtship

Tyramine can act through 3 different G protein coupled receptors: TAR1 (*honoka*, CG7485), TAR2 (*TyrR*, CG7431), and TAR3 (*TyrRII*, CG16766).[46] We used mutants in these tyramine receptor(s) to identify those mediating the feeding or courtship choice. Fed males mutant for TAR1[47] or TAR2[43] did not exhibit a behavioral phenotype (Figures 2E and 2F). However, most fed TAR3 mutants[46] and TAR2/TAR3 double mutant males[46] preferred to feed over courting a female (CPI −0.5 and −0.3, respectively) (Figures 2G and 2H). These observations were confirmed by manipulating TyrR expression in TyrR neurons using transgenic RNAi. Although downregulation of TAR1 or TAR2[46,48] in TyrR neurons did not affect the behavior of fed males (Figures 2I and 2J), knockdown of TAR3[48] in TyrR neurons promoted feeding over courtship in fed flies (Figure 2K). Therefore, activation of TyrR neurons and knockdown of TAR3 in TyrR neurons both bias behavior toward feeding. Previous studies have shown that tyramine can act as an inhibitory neuromodulator in larval and adult TyrR neurons.[43,48] Hence, tyramine is likely to inhibit TyrR neurons via TAR3, preventing feeding in sated flies (Figure 2L).

Tdc2 neurons are required for fed flies to prefer courtship

Tyramine is produced from tyrosine by the action of tyrosine decarboxylase (Tdc2) and is subsequently converted to octopamine by tyramine β-hydroxylase (TβH) (Figure 3A).[49] Octopamine and tyramine can act as independent neuromodulators, which are believed to be the fly equivalents of vertebrate adrenergic transmitters.[49] Moreover, $Tdc2^{+}$/$T\beta h^{-}$ neurons in the brain only produce tyramine and not octopamine (Figure 3G).[50,51]

To investigate a role for tyramine in the feeding-courtship choice, we evaluated the consequences of depleting tyramine using flies with a null mutation in the *Tdc2* gene ($Tdc2^{RO54}$). Most fed $Tdc2^{RO54}$ males preferred to feed over courting a female (CPI ∼−0.3 versus control ∼+0.5) (Figure 3B). Chronic or adult-specific inhibition of Tdc2 neurons in fed flies by expressing UAS-TNT or UAS-TNT;tub-$GAL80^{TS}$ under the control of Tdc2-GAL4 recapitulated the $Tdc2^{RO54}$ mutant phenotype (*Tdc2>TNT* males: CPI −0.3 versus genetic controls: ∼+0.8) (Figures 3C and 3D). Finally, we tested the effects of overexpressing Tdc2 in Tdc2 neurons in 24-h starved males. Starved *Tdc2>Tdc2* flies displayed enhanced courtship when

(C and D) Courtship preference indices of 24-h starved (C) or fed (D) *TyrR-GAL4>UAS-TrpA1* males, allowed to choose between feeding on sucrose and courting a female (n = 21–59 in C and n = 34–147 in D).

(E–H) Effects of mutating TAR receptors on behavioral choice. Courtship preference indices obtained from $TAR1^{hono}$ mutant males (n = 54–64) (E), TAR2 mutants (n = 24–31) (F), $TAR3^{\Delta 129}$ mutants (n = 53–56) (G), TAR2-$TAR3^{\Delta 124}$ double mutants (n = 25–33) (H), and wild-type controls.

(I–K) Effects of manipulating TAR receptor levels in TyrR neurons on courtship preference (n = 42–50 for *TyrR-GAL4>UAS-TAR1 RNAi*, n = 17–21 for *TyrR-GAL4>UAS-TAR2* RNAi, and n = 70–88 for *TyrR-GAL4>UAS-TAR3 RNAi*).

(L) Working model. Tyramine inhibits TyrR neurons via TAR3, preventing feeding in fed flies.

(M and N) *UAS-mCD8-GFP* expressed under the control of *TyrR-GAL4* in the adult brain (M) and VNC (N) GFP staining (green); neuropil counterstained with anti-nC82 (blue). SMP, superior medial protocerebrum; PLP, posterior lateral protocerebrum; IPS, inferior posterior slope; GNG, gnathal ganglia. Scale bars, 50 μm.

Lines show the mean and error bars indicate SEM. *p < 0.05, **p < 0.01, ***p < 0.001 calculated using Kruskal Wallis followed by Dunn's post hoc multiple comparison test for data in (A)–(D) and (I)–(K) and Mann-Whitney U test in (E)–(H). In (C) and (D), the courtship preference indices obtained from the experimental groups were compared to both genetic controls tested at activation and control temperature and to data from the same genotype at the control temperature. Absence of asterisk (*) denotes non-significant data.

See also Figure S2.

CellPress
OPEN ACCESS

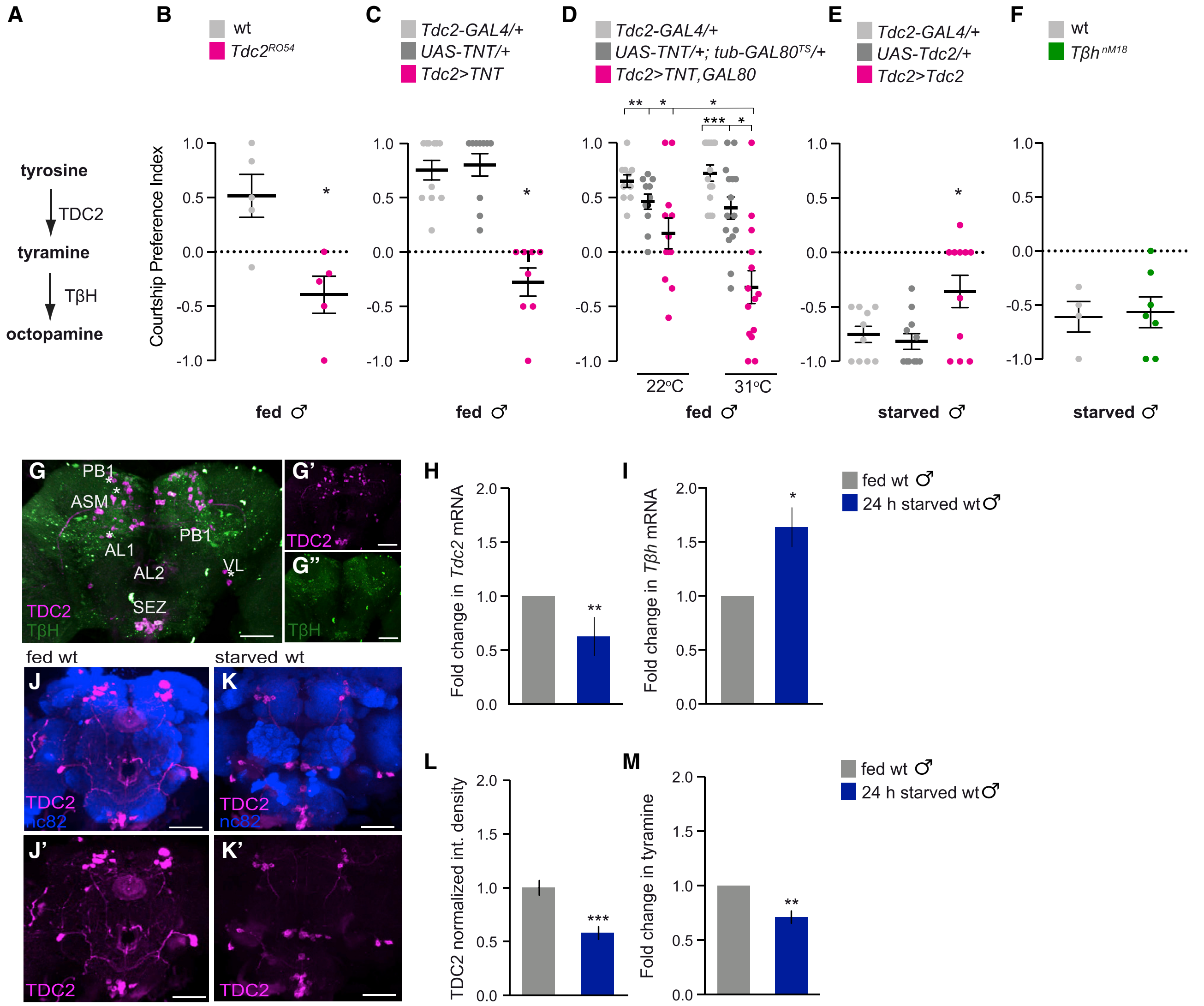

Figure 3. Tyramine biosynthesis in the brain is modulated in response to starvation

(A) The tyramine/octopamine biosynthesis pathway. TDC2, tyrosine decarboxylase; TβH, tyramine β-hydroxylase.

(B–F) Courtship preference indices obtained from starved or fed males of the indicated genotypes, choosing between feeding on sucrose and courting a female ($Tdc2^{R054}$ n = 17–20; *Tdc2-Gal4>UAS-TNT* n = 23–53; *Tdc2-GAL4>UAS-Tdc2* n = 12–24; $Tdc2^{acute}$ > TNT n = 55–80 [22°C], 61–79 [31°C]; and $T\beta h^{nM18}$ n = 17–40). *UAS-TNT/+; tub-GAL80*^{TS}*/Tdc2-GAL4* ($Tdc2^{acute}$ > *TNT*) males and genetic controls were tested at 22°C and 31°C, respectively.

(G) Representative confocal images showing TDC2 (magenta) and TβH (green) staining in a wild-type male brain. TDC2^{+}/TβH^{-} clusters are depicted with an asterisk. Anterior: ASM cluster in anterior superior medial protocerebrum, AL1 cluster in antennal lobes, VL clusters in ventrolateral region, and SEZ in sub esophageal ganglion. Posterior: PSM and PB1 clusters in posterior medial protocerebrum and SEZ in sub esophageal ganglion. Scale bar, 50 μm.

(H) A significant decrease in *Tdc2* mRNA levels (after normalization to Rp15 mRNA levels) revealed by qRT-PCR in the brains of 24-h starved males compared to those in sated males (n = 20, 4 biological replicates).

(I) A significant increase in *Tβh* mRNA levels (after normalization to Rp15 mRNA levels) revealed by qRT-PCR in brains of 24-h starved males compared to non-starved males (n = 18; 4 biological replicates).

(J–K) Representative confocal images showing nc82 (blue) and TDC2 (magenta) staining in fed (I and I′) and starved (J and J′) wild-type male brains. Scale bars, 50 μm.

(L) Integrated fluorescence of GFP under the control of *Tdc2-GAL4* normalized to average fed integrated fluorescence within each biological replicate (n = 18; 4 biological replicates).

(M) Starvation decreases tyramine levels in the fly brain. Tyramine levels were quantified using the intrinsic fluorescence of tyramine after separation by reverse-phase HPLC. Forty heads were analyzed for each condition, for each biological replicate (n = 4). The data are presented as the ratio of tyramine observed in starved/fed flies.

Lines show the mean and error bars indicate SEM. *$p < 0.05$, **$p < 0.01$, ***$p < 0.001$ calculated using Kruskal Wallis followed by Dunn's post hoc multiple comparison test for data in (C)–(E) and Mann-Whitney U test for data in (B), (F), (H), (I), (L), and (M). Absence of asterisk (*) denotes non-significant data.

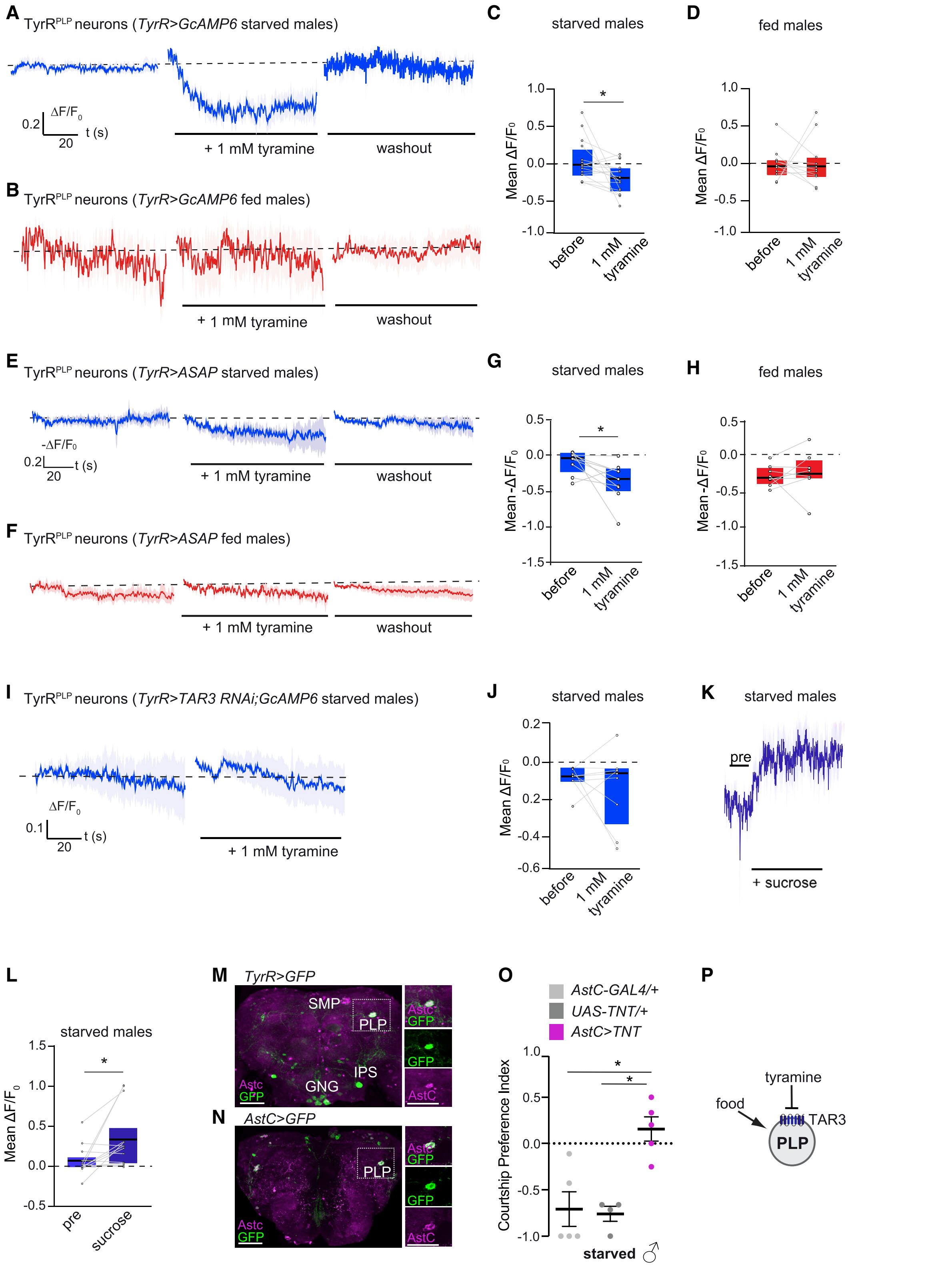

Figure 4. TyrR^{PLP} neurons respond to tyramine and sucrose in starved males

(A and B) Application of 1 mM tyramine (middle panel) leads to a decrease in the Ca^{2+} response of TyrR^{PLP} neurons only in starved (blue, A) but not fed (red, B) males, in comparison to the response before the application (left panel). After 5 min of washout with saline (right panel) responses return to baseline. The $\Delta F/F_0$

(legend continued on next page)

CellPress
OPEN ACCESS

compared with controls (CPI ~−0.4 versus genetic controls ~−0.8) (Figure 3E).

Octopamine is known to be important for inducing feeding in response to starvation.[52,53] As a result, octopamine-deficient *Tβh^nM18* mutant males[54] showed an increased latency to feed after 24 h of starvation, as compared to control flies (321.4 s versus 158.4 s, n = 19–40, respectively; $p < 0.005$, Mann-Whitney; data not shown). However, in the presence of both a food source and a female, starved *Tβh^nM18* mutant males displayed similar behavior to wild-type controls (Figure 3F). Therefore, our findings suggest that brain tyramine, but not octopamine, plays a role in the male fly's choice between initiating feeding or courtship.

Starvation decreases tyramine levels in the brain

Our findings show that either depleting flies of Tdc2 enzyme, inhibiting Tdc2-expressing neurons, or activating TyrR neurons, promoted feeding over courtship in fed males (Figures 2, 3, and S2). These results raise the possibility that high tyramine levels serve as a satiety signal in males that suppresses feeding through reduced activity of TyrR neurons. We therefore tested whether tyramine is modulated by starvation in adult flies by measuring *Tβh* and *Tdc2* expression levels in the brain after 24 h of starvation. qRT-PCR revealed a significant decrease in *Tdc2* mRNA levels of ~40% upon starvation, which would be expected to result in lower levels of tyramine in starved flies (Figure 3H). In contrast, *Tβh* mRNA levels increased by ~60% following 24 h of starvation (Figure 3I). We also quantified the immunofluorescence signal intensity in all Tdc2 clusters with an anti-TDC2 antibody, which revealed a starvation-dependent decrease of TDC2 protein levels in the fly brain (Figures 3J and 3L). Due to the lack of anti-TBH antibody, we could not compare TBH labeling in fed and starved adult flies. However, supporting our findings, TBH protein levels have been shown to increase upon starvation in *Drosophila* larvae.[48] The antagonistic effect of starvation on *Tdc2* and *Tβh* gene expression levels is likely to affect net levels of tyramine. We therefore measured tyramine in the brain of fed and starved male flies using high-performance liquid chromatography (HPLC). Consistent with gene expression levels, we detected a 30% decrease in brain tyramine upon starvation (Figure 3M). We therefore conclude that starvation decreases tyramine levels in the brain through modulation of *Tdc2* and *Tβh* gene expression, promoting feeding in flies.

TyrR^{PLP} neurons respond to tyramine and sucrose in starved males

Our data suggest that tyramine might inhibit TyrR neurons involved in the decision of whether to initiate feeding or courtship, and such modulation depends on the male's starvation levels. TyrR neurons in the brain comprise 4 clusters: the superior medial protocerebrum (SMP), the posterior lateral protocerebrum (PLP), the inferior posterior slope (IPS), and the gnathal ganglia (GNG). TyrR^{IPS} neurons have been previously implicated in courtship behavior[43] (Figure S3A). However, neither inhibiting TyrR^{IPS} neurons[55] in starved *TyrR^{IPS}>TNT* males or activating TyrR^{IPS} neurons in fed *TyrR^{IPS}>TrpA1* males affected the feeding-courtship choice (Figures S3B and S3C). Moreover, activating TyrR^{IPS} neurons did not increase food intake in fed *TyrR^{IPS}>TrpA1* males (Figure S3D). These results led us to postulate that other non-IPS TyrR neurons (TyrR$^{IPS(-)}$) are likely to mediate the behavioral choice.

We measured the effects of tyramine on TyrR$^{IPS(-)}$ neurons using live two-photon calcium imaging in fed and starved adult

represents the evoked fluorescence change from baseline. Traces were averaged from 11–16 flies. Solid line represents the mean and shaded areas indicate SEM. Genotype tested: *TyrR-GAL4/UAS-GCaMP6s;61A01-GAL80*.

(C and D) Quantification of mean $\Delta F/F_0$ responses of TyrR^{PLP} neurons over 90 s before (pre) and after the application of 1 mM tyramine in starved (blue, C) and fed (red, D) males. In starved males, a significant reduction of the mean Ca^{2+} response to 1 mM tyramine was observed (n = 16). In fed males, no changes were apparent (n = 11).

(E and F) Application of 1 mM of tyramine (middle panel) hyperpolarizes TyrR^{PLP} neurons in starved (E, blue) males, as indicated by increase in fluorescence (decrease in $-\Delta F/F_0$) of the ASAP2 voltage sensor in comparison to the response before application (left panel). In fed (F, red) males no changes were observed. The $-\Delta F/F_0$ represents the evoked fluorescence change from baseline. Traces were averaged from 8–9 flies. The voltage-imaging plot fluorescence changes are plotted on a $-\Delta F/F_0$ scale to correct for the inverse relation between membrane voltage and sensor fluorescence. Genotype tested: *UAS-ASAP2/61A01-LexA;LexAopGa80/TyR-GAL4*.

(G and H) Quantification of the mean $-\Delta F/F_0$ responses of TyrR^{PLP} neurons over 90 s before (pre) and after the application of 1 mM tyramine in starved (G, blue) and fed (H, red) males (using ASAP2). Changes to 1 mM tyramine were observed in starved (n = 9) but not fed males (n = 8).

(I) Application of 1 mM tyramine (right panel) in starved males expressing TAR3 RNAi in TyrR neurons did not change the mean Ca^{2+} response in comparison to baseline (left panel). Traces were averaged from 9 animals. Genotype tested: *UAS-GCaMP6s/+;TyR-GAL4/UAS-TAR3 RNAi*.

(J) Quantification of mean $\Delta F/F_0$ responses of TyrR^{PLP} neurons in starved males expressing TAR3 RNAi over 90 s before (pre) and after application of Tyr (1 mM tyramine). No changes were observed in the mean Ca^{2+} response to 1 mM tyramine (n = 9).

(K) In starved males, feeding on 100 mM sucrose leads to a Ca^{2+} increase in TyrR^{PLP} neurons in comparison to the response before sugar consumption (pre). Traces were averaged from 11 flies. Only flies that were confirmed to ingest sucrose were included in the analysis (n = 11). Genotype tested: *TyrR-GAL4/UAS-GCaMP6s;61A01-GAL80/+*.

(L) Quantification of mean $\Delta F/F_0$ responses of TyrR^{PLP} before (pre; 16 s) and during sugar consumption (60 s).

(M) UAS-mCD8-GFP expressed with TyrR-GAL4 in the adult brain. Anti-GFP (green) and anti-AstC (magenta) staining is shown. Magnified images of the lateral protocerebrum show clear overlap between TyrR^{PLP} cells and anti-AstC. SMP, superior medial protocerebrum; PLP, posterior lateral protocerebrum; IPS, inferior posterior slope; GNG, gnathal ganglia.

(N) UAS-mCD8-GFP expressed with *AstC-GAL4 (II)* in the adult brain. Anti-GFP and anti-AstC. Scale bars, 50 or 25 μm, insets.

(O) Courtship preference indices of 24-h starved *AstC-GAL4 (II)/UAS-TNT* males and the genetic controls (n = 26–29).

(P) Working model. TyrR^{PLP} neurons are antagonistically regulated by tyramine and food detection.

Lines show the mean and error bars indicate SEM. All boxplots represent 25th to 75th percentile and black line as the mean. *$p < 0.05$ calculated using paired t test in (C) and (D); Wilcoxon matched-pairs signed rank test in (G), (H), (J), and (L); and Kruskal Wallis test followed by Dunn's post hoc multiple comparison in (O). Absence of asterisk (*) denotes non-significant data.

See also Figure S3.

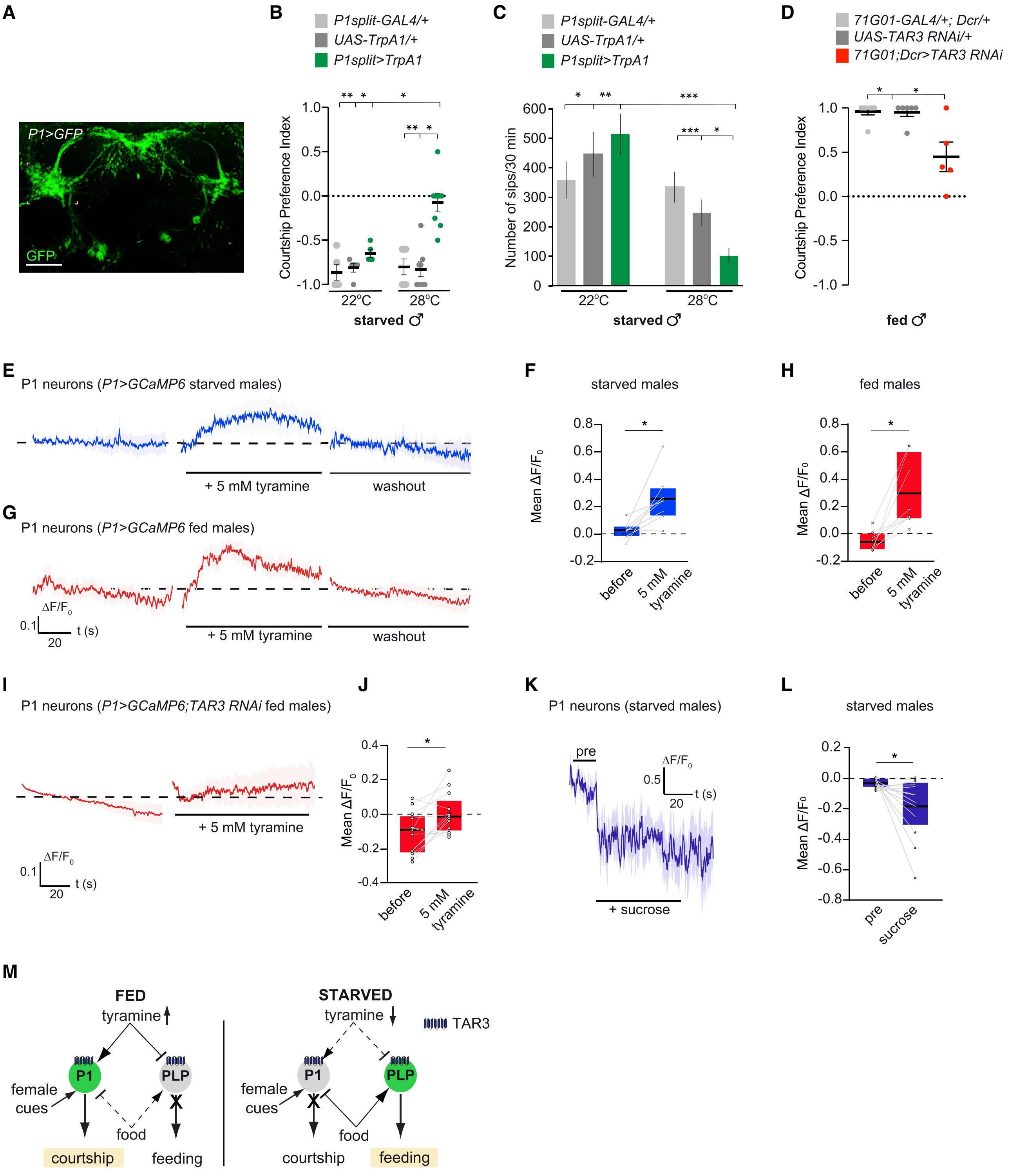

Figure 5. Tyramine and sucrose modulate the activity of P1 courtship-command neurons

(A) UAS-mCD8-GFP expressed with P1split-GAL4 in the adult brain. Green, anti-GFP. Scale bar, 50 μm.

(B) Activation of P1 neurons overrides starvation. Courtship preference index of 24-h starved *P1 split-GAL4>UAS-TrpA1* and genetic controls choosing between feeding on sucrose and courting a female measured at the activation temperature 28°C (n = 48–65) and control temperature 22°C (n = 47–74).

(C) Activation of P1 neurons decreases feeding in *P1 split-GAL4>UAS-TrpA1* starved male flies. Food intake during 30 min using flyPAD at the activation temperature 28°C (n = 65–80) and control temperature 22°C (n = 25–37).

(legend continued on next page)

male flies. We expressed the calcium indicator UAS-GCaMP6s with TyrR-GAL4 and applied exogenous tyramine to the brain of fed and starved flies. Neither $TyrR^{SMP}$ or $TyrR^{GNG}$ neurons showed a significant response to tyramine in fed or starved flies (Figures S3E and S3H). However, tyramine application induced a significant decrease in the activity of $TyrR^{PLP}$ neurons in starved males (Figures 4A and 4C). We predicted that TyrR neurons may be inactive in fed flies due to endogenous tyramine and that further inhibitory input might not change their activity. Consistent with this notion, tyramine application did not modulate $TyrR^{PLP}$ neuron activity in fed males (Figures 4B and 4D). We confirmed the inhibitory effects of tyramine in $TyrR^{PLP}$ cells in starved males by also monitoring neural activity using the genetically encoded ASAP2 voltage indicator.[56] As expected, the ASAP2 signal decreased in response to tyramine in starved but not fed males (Figures 4E–4H). Interestingly, the basal levels of ASAP2 fluorescence in $TyrR^{PLP}$ neurons were significantly higher in starved flies than in fed flies (unpaired t test, $p < 0.05$) (Figures 4E and 4F). Such a decrease in fluorescence of ASAP2 indicates that $TyrR^{PLP}$ neurons are more depolarized in starved flies. Although our behavioral data show that octopamine depletion does not significantly affect the choice between feeding and courtship (Figure 3), we also verified that octopamine application did not stimulate a significant Ca^{2+} response in $TyrR^{PLP}$ (Figures S3I and S3J) or any other TyrR clusters (data not shown).

Our behavioral findings argue that tyramine may inhibit TyrR neurons via the TAR3 receptor, reducing feeding in sated flies (Figure 2). We therefore tested whether RNAi-mediated downregulation of TAR3 altered the effects of tyramine on $TyrR^{PLP}$ neurons in Ca^{2+} imaging. Indeed, decreasing TAR3 expression in TyrR neurons abolished the inhibitory effect of tyramine on $TyrR^{PLP}$ neural activity (Figures 4I and 4J), further supporting a role for TAR3 in the feeding-courtship choice.

Because our results indicate that TyrR neurons promote feeding and suppress courtship when a food source becomes available (Figures 2, 4, and S2), we reasoned that $TyrR^{PLP}$ neurons might be additionally modulated by feeding. To explore this idea, we monitored GCaMP6 fluorescence in $TyrR^{PLP}$ neurons while 24-h starved flies were offered a drop of 100 mM sucrose. $TyrR^{PLP}$ neurons did not respond to water (data not shown) but they showed a spike in GCaMP6 signal in response to sucrose ingestion (Figures 4K and 4L). Therefore, $TyrR^{PLP}$ neurons are oppositely modulated by tyramine levels and food signals.

To further investigate the involvement of $TyrR^{PLP}$ neurons in the feeding-courtship choice, we searched for GAL4 lines that may target these neurons. We noted that the allatostatin C (AstC) neuropeptide is expressed in neurons near the posterior medial protocerebrum (PMP), which include two notably large bilaterally symmetrical neurons that resembled $TyrR^{PLP}$ neurons.[57] We confirmed these are the same $TyrR^{PLP}$ neurons by co-labeling brains with TyrR-GAL4 driven mGFP and an anti-AstC antibody (Figures 4M and 4N). We therefore next used two AstC-GAL4 drivers to block $TyrR^{PLP}$ and other $AstC^{+}$ neurons with UAS-TNT. Starved *AstC>TNT* males significantly favored courtship over feeding, compared to controls (CPI: $\sim$+0.1 versus controls ~ -0.7 and ~ -0.9; Figures 4O and S3L), supporting a role for $TyrR^{PLP}$ neurons in the behavioral choice. We conclude that $TyrR^{PLP}$ neurons integrate both starvation levels—via tyramine levels—and food availability to regulate the fly's choice between initiating courtship or feeding (Figure 4P).

Tyramine and sucrose modulate the activity of courtship-command P1 neurons

P1 neurons are a central node that initiates male courtship in *Drosophila* (Figure 5A).[21,28] We thus tested whether P1 cells play a role in the choice between feeding and courtship. Artificial activation of P1 neurons using UAS-TrpA1 at 28°C increased preference for courtship when starved males were given the choice between eating or courting (CPI $\sim$0 versus genetic controls: -0.8 (28°C); and -0.7 at 22°C) (Figure 5B).

We also found that P1 activation reduced food intake in starved flies (Figure 5C), indicating that tyramine might also act on P1 neurons to influence the behavioral choice. We therefore assessed whether exogenous tyramine modulated P1 neuron activity using live imaging in starved and fed *71G01>GcAMP6* males. In contrast to the inhibition observed in $TyrR^{PLP}$ neurons,

(D) Knockdown of TAR3 receptor in P1 neurons alters behavioral choice. Courtship preference index of fed *71G01-GAL4;Dcr>UAS-TAR3 RNAi* males and genetic controls choosing between feeding on sucrose and courting a female (n = 18–40).

(E and G) Application of 5 mM tyramine (middle panel) leads to an increase in the Ca^{2+} response of P1 neurons in both starved (E, blue) and fed (G, red) males, in comparison to the response before application (left panel). After 5 min of washout with saline (right panel) the responses return to baseline. Traces were averaged from 8 flies. Genotype tested: *71G01-GAL4/UAS-GCaMP6s*.

(F and H) Quantification of the mean ΔF/F0 responses of P1 neurons over 90 s before (pre) and after application of 5 mM tyramine in starved (F, blue) and fed (H, red) males. Starved (n = 8) and fed (n = 8) males respond to the application of 5 mM tyramine with an increase in the mean Ca^{2+} response.

(I) Application of 5 mM tyramine (right panel) in fed males expressing the TAR3 RNAi under the control of 71G01-GAL4 changed the mean Ca^{2+} response in comparison to the baseline (left panel). Genotype tested: *UAS-GCaMP6s/+;UAS-TAR3 RNAi/71G01-GAL4*.

(J) Quantification of mean $\Delta F/F_0$ responses of P1 neurons in starved males expressing TAR3 RNAi over 90 s before (pre) and after application of Tyr (5 mM tyramine). A significant increase of the mean Ca^{2+} response to 5 mM tyramine was observed (n = 13).

(K) In starved males, sucrose (100 mM) ingestion leads to a Ca^{2+} decrease in P1 neurons (n = 13). Traces were averaged from 13 flies. Only animals that were confirmed to ingest sucrose were included in the analysis. Genotype tested: *71G01-GAL4/UAS-GCaMP6s*.

(L) Quantification of mean $\Delta F/F_0$ responses of P1 neurons before (pre; 16 s) and during sugar consumption (60 s).

(M) Feeding-courtship behavioral choice working model. Reduced levels of tyramine in starved males leads to increased TyrR neural activity (e.g., $TyrR^{PLP}$). These neurons are further activated upon ingestion of a food source, promoting feeding over courtship. P1 courtship-promoting neurons are antagonistically modulated by tyramine and sucrose, favoring courtship over feeding in fed flies.

Scatterplots show courtship preference indices obtained from different groups of flies treated as independent biological replicates. Lines show the mean and error bars indicate SEM. All boxplots represent 25th to 75th percentile and black line as the mean. *$p < 0.05$, **$p < 0.01$, ***$p < 0.001$ calculated using Kruskal Wallis test followed by Dunn's post hoc multiple comparison in (B)–(D); paired t test in (F), (H), and (J); and Wilcoxon matched-pairs signed rank test in (L). Absence of asterisk (*) denotes non-significant data.

tyramine activated P1 neurons in both starved and sated flies (Figures 5E–5H). Because TAR3 mediates the response in TyrR^{PLP} neurons (Figure 2), we tested whether tyramine modulates P1 neurons via TAR3. Due to the lack of TAR3 antibody, we could not visualize TAR3 protein specifically in P1 neurons. However, previous single-cell transcriptomic analyses have shown that *fruitless*-expressing neurons, which include P1 cells, express the *Tar3* gene.[58] Consistent with this observation, RNAi-mediated knockdown of TAR3 in P1 and a few other cells using 71G01-GAL4[6,59] decreased courtship preference in fed males (CPI 0.4 versus controls ~1) (Figure 5D). Moreover, *71G01>TAR3 RNAi* flies showed a decreased response in P1 neurons when locally applying tyramine in Ca^{2+} imaging experiments (Cliff's delta; 0.396 versus 0.969) (Figures 5G and 5H versus Figures 5I and 5J). Hence, our data suggest that TAR3 plays a role in the tyramine-dependent modulation of P1 neurons, likely influencing the choice between courtship and feeding. However, because TAR3 RNAi in P1 neurons did not completely abolish the response to tyramine in Ca^{2+} imaging experiments, we cannot exclude the role of other tyramine receptors in P1-mediated behavioral choice.

We last tested if P1 neurons are modulated by food ingestion, like TyrR neurons. Interestingly, sucrose ingestion inhibited P1 neurons in starved males (Figures 5K and 5L), as opposed to TyrR neurons that were activated by the fly detecting sugar (Figures 4K and 4L).

Taken together, our results demonstrate that feeding-promoting TyrR neurons and courtship-promoting P1 neurons are antagonistically modulated by tyramine and food, which likely coordinates the fly's behavior to reflect its internal state and external resource availability (Figure 5M).

DISCUSSION

When exposed to competing stimuli and needs, animals are forced to select one action at the expense of others. Although the "singleness of action" principle was formulated by Sherrington in 1906,[2] how alternative behaviors compete for expression in the nervous system remains unknown. Here, we established a scenario where *Drosophila* fruit flies must choose between the two essential drives of mating or feeding.

We found that courtship is overridden by starvation in sexually naive male flies that have been starved for 24 h. This is consistent with Maslow's hierarchy of needs theory, which postulates that the most basic physiological needs (e.g., food, water, and sleep) must be fulfilled before higher needs can be met (e.g., mating and cooperation).[60]

An increasing number of studies suggest that prioritization of feeding could be a ubiquitous behavioral strategy in the animal kingdom. For instance, feeding has been shown to inhibit escape responses in invertebrates[3] and can dominate rival motivational drives such as thirst, anxiety, innate fear, and social interaction in mammals.[61–63] Yet behavioral prioritization is plastic and context-dependent. The decision to feed can be reshaped by manipulating the reproductive and feeding drives of the fly, as well as by the quality of the available food source. Moreover, as caloric need increases, flies switched their initial behavioral preference from courtship to feeding. We found a significant behavioral shift following 15 h of starvation, suggesting that this might be the tipping point where feeding becomes more urgent than reproduction.

Maximizing resource availability is central to animal fitness. Our findings raise the interesting possibility that flies might implement a behavioral strategy to maximize the resources present in their surroundings. First, males are quicker at initiating feeding when there is a female around. Second, after feeding, most males switch to courtship without further delay, sometimes within seconds. The ability to navigate and exploit natural environments largely depends on remembering locations. For example, mice recall the location of shelters and use this information not only to escape a threat but also to choose a defensive strategy.[64] Flies can use visual landmarks for spatial memory in specific contexts[65,66] and rely on idiothetic cues to find a food source.[20,67] Additional studies should reveal how the fly brain generates a spatial map that allows them to be constantly aware of their surroundings and adapt to acute changes.

The expression of feeding and courtship behaviors appear to be mutually exclusive in flies, raising the question as to how flies resolve which behavior to express. Neuromodulators, like biogenic amines, are crucial for engendering circuit flexibility, enabling adaptations to physiological states and external context.[68] Our study identified the tyramine pathway as an important mediator in the choice between sex and feeding. We demonstrate that tyramine in the brain is regulated by nutritional status. Consistent with previous work in *Drosophila* larvae,[48] we show that starvation modulates Tdc2 and Tβh gene expression, resulting in decreased tyramine in the brain. In line with this, brain tyrosine has been shown to decrease upon starvation in rats, reducing the available substrate for tyramine production.[69]

Our findings show that starved males court as efficiently as fed males when no food is around, and these males robustly suppress courtship only when nutritious food is accessible. In rodents, hunger suppresses competing motivational drives more efficiently when food becomes accessible.[61,62] Therefore, suitable resource availability appears to be an essential feature of behavioral prioritization. In line with this, we found TyrR^{PLP} neurons get activated and P1 neurons get inhibited upon sucrose ingestion, likely sustaining the feeding response in starved flies.

Taken together, our findings suggest that high tyramine activates courtship-promoting P1 neurons and inhibits feeding-promoting TyrR neurons, favoring courtship over feeding initiation in fed flies. Upon starvation, low tyramine releases inhibition on TyrR neurons and reduces P1 activity, shifting the balance toward feeding. Cues signaling quality food would further activate TyrR neurons and decrease P1 neural activity, reinforcing the feeding response. It will be interesting to determine how cues conveying food availability and quality are integrated in P1 and TyrR cells to bias behavioral outcome. Flies can discriminate between nutritional sugars and their non-nutritional enantiomers.[38,70] Notably, Qi et al.[71] showed that flies sense the nutritional value of a food source as quickly as 20-s post-ingestion, through a mechanism yet unknown.

Future investigation awaits identifying how different starvation and satiety signals are timely delivered and act in concert to modulate Tdc2 tyramine-producing neurons. Dedicated peripheral tissues and central nutrient sensors in flies detect

and respond to perturbations in macronutrient homeostasis by the release of specific endocrine and neuromodulatory signals. These include insulin-like peptides (DILPs), the insect analog of mammalian glucagon (AKH), and the homolog of mammalian neuropeptide Y (dNPF).[36] The mechanism by which energy deficits are represented in tyraminergic neurons is poorly understood. Moreover, how nutritional state may induce changes in biogenic amine synthesis and release remains to be determined.

Tyramine signaling has also been shown to play a role in hunger-dependent threat-reward decision-making in *C. elegans*, suggesting conservation of components of decision-making circuits across animal species.[72] Norepinephrine, a functional analog of tyramine in vertebrates, has been shown to modulate food intake[73,74] and some aspects of reproductive behaviors.[75,76] Similar neuronal pathways controlled by biogenic amines may facilitate behavioral choices in mammals.

STAR★METHODS

Detailed methods are provided in the online version of this paper and include the following:

- KEY RESOURCES TABLE
- RESOURCE AVAILABILITY
  - Lead contact
  - Materials availability
  - Data and code availability
- EXPERIMENTAL MODEL AND SUBJECT DETAILS
- METHOD DETAILS
  - Behavioral assays
  - Behavioral Choice Assay
  - Courtship Preference Index (CPI)
  - Courtship and feeding assays
  - Direct and Indirect switch
  - FlyPAD feeding assays
  - Thermogenetic neuronal activation
  - TARGET system silencing experiments
  - qRT-PCR
  - Immunohistochemistry and fluorescence intensity quantification
  - Two-photon Calcium Imaging
  - HPLC
- QUANTIFICATION AND STATISTICAL ANALYSIS

SUPPLEMENTAL INFORMATION

Supplemental information can be found online at https://doi.org/10.1016/j.cub.2021.07.029.

ACKNOWLEDGMENTS

We thank Andrew Lin, Nuria Romero, and Anissa Kempf for insightful comments and critical assessment of the manuscript. We are grateful to Jan A. Veenstra, Edward Blumenthal, Sarah Certel, Henrike Scholz, Carsten Duch, Thomas Preat, Pierre-Yves Plaçais, David Anderson, Kim Young-Joon, and Yi Rao for sharing fly stocks or antibodies with us. We thank Alessandro di Maio and the Birmingham Advanced Light Microscopy (BALM) facility for support with confocal microscopy. We would also like to thank Raksha Gohel for help with fly collection and immunohistochemistry, Anand Singh and Saverio Brogna for help with qRT-PCR experiments, Budhaditya Choudhary for help with tracing the trajectories of behaving flies, and Laurie Cazale-Debat for the schematics. Finally, we thank members of the Rezaval lab for useful comments on the manuscript. This work was supported by BBSRC (BB/S009299/1), Wellcome Trust (214062/Z/18/Z), and Royal Society Research (RGS\R2\180272) to C.R.; a Darwin studentship to S.R.; and a BBSRC David Phillips fellowship to P.J.M.

AUTHOR CONTRIBUTIONS

Conceptualization, S.J.C., S.R., and C.R.; Methodology, S.J.C., S.R., P.F.J., L.A.B., S.G., J.M., M.W., P.J.M., S.W., and C.R.; Investigation, J.C., S.R., P.F.J., L.A.B., S.G., J.M., M.W., P.J.M., and C.R.; Resources, C.R.; Writing, S.J.C., S.R., P.F.J., S.W., and C.R.; Supervision, C.R.; Funding Acquisition, C.R.

DECLARATION OF INTERESTS

The authors declare no competing interests.

Received: December 10, 2020
Revised: May 25, 2021
Accepted: July 13, 2021
Published: August 5, 2021

REFERENCES

1. Calabrese, R.L. (2003). Behavioral choices: how neuronal networks make decisions. Curr. Biol. *13*, R140–R142.
2. Sherrington, C. (1906). The Integrative Action of the Nervous System (Yale University Press).
3. Kristan, W.B. (2008). Neuronal decision-making circuits. Curr. Biol. *18*, R928–R932.
4. Kovac, M.P., and Davis, W.J. (1980). Neural mechanism underlying behavioral choice in Pleurobranchaea. J. Neurophysiol. *43*, 469–487.
5. Mann, K., Gordon, M.D., and Scott, K. (2013). A pair of interneurons influences the choice between feeding and locomotion in Drosophila. Neuron *79*, 754–765.
6. Hoopfer, E.D., Jung, Y., Inagaki, H.K., Rubin, G.M., and Anderson, D.J. (2015). P1 interneurons promote a persistent internal state that enhances inter-male aggression in Drosophila. eLife *4*, e11346.
7. Chen, J., Reiher, W., Hermann-Luibl, C., Sellami, A., Cognigni, P., Kondo, S., Helfrich-Förster, C., Veenstra, J.A., and Wegener, C. (2016). Allatostatin A signalling in Drosophila regulates feeding and sleep and is modulated by PDF. PLoS Genet. *12*, e1006346.
8. Chen, D., Sitaraman, D., Chen, N., Jin, X., Han, C., Chen, J., Sun, M., Baker, B.S., Nitabach, M.N., and Pan, Y. (2017). Genetic and neuronal mechanisms governing the sex-specific interaction between sleep and sexual behaviors in Drosophila. Nat. Commun. *8*, 154.
9. Senapati, B., Tsao, C.H., Juan, Y.A., Chiu, T.H., Wu, C.L., Waddell, S., and Lin, S. (2019). A neural mechanism for deprivation state-specific expression of relevant memories in Drosophila. Nat. Neurosci. *22*, 2029–2039.
10. Murakami, K., Yurgel, M.E., Stahl, B.A., Masek, P., Mehta, A., Heidker, R., Bollinger, W., Gingras, R.M., Kim, Y.J., Ja, W.W., et al. (2016). translin is required for metabolic regulation of sleep. Curr. Biol. *26*, 972–980.
11. McDonald, D.M., and Keene, A.C. (2010). The sleep-feeding conflict: understanding behavioral integration through genetic analysis in Drosophila. Aging (Albany NY) *2*, 519–522.
12. Murphy, K.R., Deshpande, S.A., Yurgel, M.E., Quinn, J.P., Weissbach, J.L., Keene, A.C., Dawson-Scully, K., Huber, R., Tomchik, S.M., and Ja, W.W. (2016). Postprandial sleep mechanics in *Drosophila*. eLife *5*, e19334.
13. Gorter, J.A., Jagadeesh, S., Gahr, C., Boonekamp, J.J., Levine, J.D., and Billeter, J.C. (2016). The nutritional and hedonic value of food modulate sexual receptivity in Drosophila melanogaster females. Sci. Rep. *6*, 19441.

14. Chapman, T., and Partridge, L. (1996). Female fitness in Drosophila melanogaster: an interaction between the effect of nutrition and of encounter rate with males. Proc. Biol. Sci. *263*, 755–759.
15. Fricke, C., Bretman, A., and Chapman, T. (2010). Female nutritional status determines the magnitude and sign of responses to a male ejaculate signal in Drosophila melanogaster. J. Evol. Biol. *23*, 157–165.
16. Good, T.P., and Tatar, M. (2001). Age-specific mortality and reproduction respond to adult dietary restriction in Drosophila melanogaster. J. Insect Physiol. *47*, 1467–1473.
17. Lebreton, S., Trona, F., Borrero-Echeverry, F., Bilz, F., Grabe, V., Becher, P.G., Carlsson, M.A., Nässel, D.R., Hansson, B.S., Sachse, S., and Witzgall, P. (2015). Feeding regulates sex pheromone attraction and courtship in Drosophila females. Sci. Rep. *5*, 13132.
18. Vargas, M.A., Luo, N., Yamaguchi, A., and Kapahi, P. (2010). A role for S6 kinase and serotonin in postmating dietary switch and balance of nutrients in D. melanogaster. Curr. Biol. *20*, 1006–1011.
19. Ribeiro, C., and Dickson, B.J. (2010). Sex peptide receptor and neuronal TOR/S6K signaling modulate nutrient balancing in Drosophila. Curr. Biol. *20*, 1000–1005.
20. Corrales-Carvajal, V.M., Faisal, A.A., and Ribeiro, C. (2016). Internal states drive nutrient homeostasis by modulating exploration-exploitation trade-off. eLife *5*, e19920.
21. Ellendersen, B.E., and von Philipsborn, A.C. (2017). Neuronal modulation of D. melanogaster sexual behaviour. Curr. Opin. Insect Sci. *24*, 21–28.
22. Agrawal, S., Safarik, S., and Dickinson, M. (2014). The relative roles of vision and chemosensation in mate recognition of Drosophila melanogaster. J. Exp. Biol. *217*, 2796–2805.
23. Spieth, H.T. (1966). Drosophilid mating behavior: the behaviour of decapitated females. Anim. Behav. *14*, 226–235.
24. Somashekar, K., Krishna, M.S., Hegde, S.N., and Jayaramu, S.C. (2011). Effects of age on female reproductive success in Drosophila bipectinata. J. Insect Sci. *11*, 132.
25. Bastock, M., and Manning, A. (1955). The courtship of Drosophila melanogaster. Behaviour *8*, 85–110.
26. Zer-Krispil, S., Zak, H., Shao, L., Ben-Shaanan, S., Tordjman, L., Bentzur, A., Shmueli, A., and Shohat-Ophir, G. (2018). Ejaculation induced by the activation of Crz neurons is rewarding to Drosophila males. Curr. Biol. *28*, 1445–1452.e3.
27. Clowney, E.J., Iguchi, S., Bussell, J.J., Scheer, E., and Ruta, V. (2015). Multimodal chemosensory circuits controlling male courtship in Drosophila. Neuron *87*, 1036–1049.
28. Kohatsu, S., Koganezawa, M., and Yamamoto, D. (2011). Female contact activates male-specific interneurons that trigger stereotypic courtship behavior in Drosophila. Neuron *69*, 498–508.
29. Kallman, B.R., Kim, H., and Scott, K. (2015). Excitation and inhibition onto central courtship neurons biases Drosophila mate choice. eLife *4*, e11188.
30. Kohatsu, S., and Yamamoto, D. (2015). Visually induced initiation of Drosophila innate courtship-like following pursuit is mediated by central excitatory state. Nat. Commun. *6*, 6457.
31. von Philipsborn, A.C., Liu, T., Yu, J.Y., Masser, C., Bidaye, S.S., and Dickson, B.J. (2011). Neuronal control of Drosophila courtship song. Neuron *69*, 509–522.
32. Kimura, K., Hachiya, T., Koganezawa, M., Tazawa, T., and Yamamoto, D. (2008). Fruitless and doublesex coordinate to generate male-specific neurons that can initiate courtship. Neuron *59*, 759–769.
33. Zhang, S.X., Miner, L.E., Boutros, C.L., Rogulja, D., and Crickmore, M.A. (2018). Motivation, perception, and chance converge to make a binary decision. Neuron *99*, 376–388.e6.
34. Zhang, S.X., Rogulja, D., and Crickmore, M.A. (2016). Dopaminergic circuitry underlying mating drive. Neuron *91*, 168–181.
35. Itskov, P.M., and Ribeiro, C. (2013). The dilemmas of the gourmet fly: the molecular and neuronal mechanisms of feeding and nutrient decision making in Drosophila. Front. Neurosci. *7*, 12.
36. Lin, S., Senapati, B., and Tsao, C.-H. (2019). Neural basis of hunger-driven behaviour in Drosophila. Open Biol. *9*, 180259.
37. Itskov, P.M., Moreira, J.M., Vinnik, E., Lopes, G., Safarik, S., Dickinson, M.H., and Ribeiro, C. (2014). Automated monitoring and quantitative analysis of feeding behaviour in Drosophila. Nat. Commun. *5*, 4560.
38. Dus, M., Ai, M., and Suh, G.S. (2013). Taste-independent nutrient selection is mediated by a brain-specific Na+ /solute co-transporter in Drosophila. Nat. Neurosci. *16*, 526–528.
39. Brand, A.H., and Perrimon, N. (1993). Targeted gene expression as a means of altering cell fates and generating dominant phenotypes. Development *118*, 401–415.
40. Sweeney, S.T., Broadie, K., Keane, J., Niemann, H., and O'Kane, C.J. (1995). Targeted expression of tetanus toxin light chain in Drosophila specifically eliminates synaptic transmission and causes behavioral defects. Neuron *14*, 341–351.
41. McGuire, S.E., Le, P.T., Osborn, A.J., Matsumoto, K., and Davis, R.L. (2003). Spatiotemporal rescue of memory dysfunction in Drosophila. Science *302*, 1765–1768.
42. Hamada, F.N., Rosenzweig, M., Kang, K., Pulver, S.R., Ghezzi, A., Jegla, T.J., and Garrity, P.A. (2008). An internal thermal sensor controlling temperature preference in Drosophila. Nature *454*, 217–220.
43. Huang, J., Liu, W., Qi, Y.X., Luo, J., and Montell, C. (2016). Neuromodulation of courtship drive through tyramine-responsive neurons in the Drosophila brain. Curr. Biol. *26*, 2246–2256.
44. Rezával, C., Pattnaik, S., Pavlou, H.J., Nojima, T., Brüggemeier, B., D'Souza, L.A.D., Dweck, H.K.M., and Goodwin, S.F. (2016). Activation of latent courtship circuitry in the brain of Drosophila females induces male-like behaviors. Curr. Biol. *26*, 2508–2515.
45. Asahina, K., Watanabe, K., Duistermars, B.J., Hoopfer, E., González, C.R., Eyjólfsdóttir, E.A., Perona, P., and Anderson, D.J. (2014). Tachykinin-expressing neurons control male-specific aggressive arousal in Drosophila. Cell *156*, 221–235.
46. Zhang, H., and Blumenthal, E.M. (2017). Identification of multiple functional receptors for tyramine on an insect secretory epithelium. Sci. Rep. *7*, 168.
47. Arakawa, S., Gocayne, J.D., McCombie, W.R., Urquhart, D.A., Hall, L.M., Fraser, C.M., and Venter, J.C. (1990). Cloning, localization, and permanent expression of a Drosophila octopamine receptor. Neuron *4*, 343–354.
48. Schützler, N., Girwert, C., Hügli, I., Mohana, G., Roignant, J.-Y., Ryglewski, S., and Duch, C. (2019). Tyramine action on motoneuron excitability and adaptable tyramine/octopamine ratios adjust *Drosophila* locomotion to nutritional state. Proc. Natl. Acad. Sci. USA *116*, 3805–3810.
49. Roeder, T. (2005). Tyramine and octopamine: ruling behavior and metabolism. Annu. Rev. Entomol. *50*, 447–477.
50. Busch, S., Selcho, M., Ito, K., and Tanimoto, H. (2009). A map of octopaminergic neurons in the Drosophila brain. J. Comp. Neurol. *513*, 643–667.
51. Burke, C.J., Huetteroth, W., Owald, D., Perisse, E., Krashes, M.J., Das, G., Gohl, D., Silies, M., Certel, S., and Waddell, S. (2012). Layered reward signalling through octopamine and dopamine in Drosophila. Nature *492*, 433–437.
52. Youn, H., Kirkhart, C., Chia, J., and Scott, K. (2018). A subset of octopaminergic neurons that promotes feeding initiation in Drosophila melanogaster. PLoS ONE *13*, e0198362.
53. Li, Y., Hoffmann, J., Li, Y., Stephano, F., Bruchhaus, I., Fink, C., and Roeder, T. (2016). Octopamine controls starvation resistance, life span and metabolic traits in Drosophila. Sci. Rep. *6*, 35359.
54. Monastirioti, M., Linn, C.E., Jr., and White, K. (1996). Characterization of Drosophila tyramine beta-hydroxylase gene and isolation of mutant flies lacking octopamine. J. Neurosci. *16*, 3900–3911.
55. Scheunemann, L., Plaçais, P.-Y., Dromard, Y., Schwärzel, M., and Preat, T. (2018). Dunce phosphodiesterase acts as a checkpoint for Drosophila long-term memory in a pair of serotonergic neurons. Neuron *98*, 350–365.

CellPress
OPEN ACCESS

56. St-Pierre, F., Marshall, J.D., Yang, Y., Gong, Y., Schnitzer, M.J., and Lin, M.Z. (2014). High-fidelity optical reporting of neuronal electrical activity with an ultrafast fluorescent voltage sensor. Nat. Neurosci. *17*, 884–889.
57. Díaz, M.M., Schlichting, M., Abruzzi, K.C., Long, X., and Rosbash, M. (2019). Allatostatin-C/AstC-R2 is a novel pathway to modulate the circadian activity pattern in Drosophila. Curr. Biol. *29*, 13–22.e3.
58. Davie, K., Janssens, J., Koldere, D., De Waegeneer, M., Pech, U., Kreft, Ł., Aibar, S., Makhzami, S., Christiaens, V., Bravo González-Blas, C., et al. (2018). A single-cell transcriptome atlas of the aging Drosophila brain. Cell *174*, 982–998.e20.
59. Zhou, C., Pan, Y., Robinett, C.C., Meissner, G.W., and Baker, B.S. (2014). Central brain neurons expressing doublesex regulate female receptivity in Drosophila. Neuron *83*, 149–163.
60. Maslow, A.H. (1943). A theory of human motivation. Psychol. Rev. *50*, 26.
61. Burnett, C.J., Funderburk, S.C., Navarrete, J., Sabol, A., Liang-Guallpa, J., Desrochers, T.M., and Krashes, M.J. (2019). Need-based prioritization of behavior. eLife *8*, e44527.
62. Burnett, C.J., Li, C., Webber, E., Tsaousidou, E., Xue, S.Y., Brüning, J.C., and Krashes, M.J. (2016). Hunger-driven motivational state competition. Neuron *92*, 187–201.
63. Sutton, A.K., and Krashes, M.J. (2020). Integrating hunger with rival motivations. Trends Endocrinol. Metab. *31*, 495–507.
64. Vale, R., Evans, D.A., and Branco, T. (2017). Rapid spatial learning controls instinctive defensive behavior in mice. Curr. Biol. *27*, 1342–1349.
65. Ofstad, T.A., Zuker, C.S., and Reiser, M.B. (2011). Visual place learning in Drosophila melanogaster. Nature *474*, 204–207.
66. Neuser, K., Triphan, T., Mronz, M., Poeck, B., and Strauss, R. (2008). Analysis of a spatial orientation memory in Drosophila. Nature *453*, 1244–1247.
67. Kim, I.S., and Dickinson, M.H. (2017). Idiothetic path integration in the fruit fly Drosophila melanogaster. Curr. Biol. *27*, 2227–2238.e3.
68. Nusbaum, M.P., and Blitz, D.M. (2012). Neuropeptide modulation of microcircuits. Curr. Opin. Neurobiol. *22*, 592–601.
69. Schweiger, U., Warnhoff, M., and Pirke, K.-M. (1985). Brain tyrosine availability and the depression of central nervous norepinephrine turnover in acute and chronic starvation in adult male rats. Brain Res. *335*, 207–212.
70. Burke, C.J., and Waddell, S. (2011). Remembering nutrient quality of sugar in Drosophila. Curr. Biol. *21*, 746–750.
71. Qi, W., Yang, Z., Lin, Z., Park, J.-Y., Suh, G.S.B., and Wang, L. (2015). A quantitative feeding assay in adult Drosophila reveals rapid modulation of food ingestion by its nutritional value. Mol. Brain *8*, 87.
72. Ghosh, D.D., Sanders, T., Hong, S., McCurdy, L.Y., Chase, D.L., Cohen, N., Koelle, M.R., and Nitabach, M.N. (2016). Neural architecture of hunger-dependent multisensory decision making in C. elegans. Neuron *92*, 1049–1062.
73. Leibowitz, S.F., Hammer, N.J., and Chang, K. (1983). Feeding behavior induced by central norepinephrine injection is attenuated by discrete lesions in the hypothalamic paraventricular nucleus. Pharmacol. Biochem. Behav. *19*, 945–950.
74. Shor-Posner, G., Grinker, J.A., Marinescu, C., and Leibowitz, S.F. (1985). Role of hypothalamic norepinephrine in control of meal patterns. Physiol. Behav. *35*, 209–214.
75. McIntosh, T.K., and Barfield, R.J. (1984). Brain monoaminergic control of male reproductive behavior. III. Norepinephrine and the post-ejaculatory refractory period. Behav. Brain Res. *12*, 275–281.
76. Murphy, L.L., Gher, J., Steger, R.W., and Bartke, A. (1994). Effects of delta 9-tetrahydrocannabinol on copulatory behavior and neuroendocrine responses of male rats to female conspecifics. Pharmacol. Biochem. Behav. *48*, 1011–1017.
77. Friard, O., and Gamba, M. (2016). BORIS: a free, versatile open-source event-logging software for video/audio coding and live observations. Methods Ecol. Evol. *7*, 1325–1330.
78. Pologruto, T.A., Sabatini, B.L., and Svoboda, K. (2003). ScanImage: flexible software for operating laser scanning microscopes. Biomed. Eng. Online *2*, 13.
79. Schindelin, J., Arganda-Carreras, I., Frise, E., Kaynig, V., Longair, M., Pietzsch, T., Preibisch, S., Rueden, C., Saalfeld, S., Schmid, B., et al. (2012). Fiji: an open-source platform for biological-image analysis. Nat. Methods *9*, 676–682.

STAR★METHODS

KEY RESOURCES TABLE

REAGENT OR RESOURCE	SOURCE	IDENTIFIER
Antibodies		
Anti-Bruchpilot (Brp), nc82	Developmental Studies Hybridoma Bank	RRID: AB_528108
Rabbit anti-Tdc2	Fisher	GR3319265-1
anti-TβH rat	Gift from Maria Monastirioti	N/A
Chicken anti-GFP	Abcam	ab92456
Rabbit anti-AstC	Gift from Jan Veenstra	N/A
633 goat anti-mouse	ThermoFisher Scientific	A21050
546 goat anti-rabbit	ThermoFisher Scientific	A11035
488 goat anti-chicken	ThermoFisher Scientific	A11039
633 goat anti-rabbit	ThermoFisher Scientific	35563
594 goat anti-rat	ThermoFisher Scientific	A11007
Experimental models: Organisms/strains		
Canton-S	Bloomington DSC	RRID: BDSC_64349
Tdc2-GAL4	Bloomington DSC	RRID: BDSC 9313
UAS-TNT	Bloomington DSC	RRID: BDSC_28837
UAS-dTrpA1	Bloomington DSC	RRID: BDSC_26264
UAS-mCD8::GFP	Bloomington DSC	RRID: BDSC_32185
LexAopGal80	Bloomington DSC	RRID: BDSC_32214
UAS-TAR1 RNAi	Bloomington DSC	RRID: BDSC_28332
UAS-TAR2 RNAi	Bloomington DSC	RRID: BDSC_25857
UAS-TAR3 RNAi	Bloomington DSC	RRID: BDSC_27670
UAS-Tdc2	Bloomington DSC	RRID: BDSC_9316
71G01-LexA	Bloomington DSC	RRID: BDSC_54733
71G01-GAL4	Bloomington DSC	RRID: BDSC_39599
UAS-ASAP2	Bloomington DSC	RRID: BDSC_65414
TyrR-GAL4	Bloomington DSC	RRID: BDSC_67129
UAS-GCaMP6s	Bloomington DSC	RRID: BDSC_42749
UAS > stop > TNT	Gift from Barry Dickson[31]	N/A
Otd-FLP	Gift from David Anderson[45]	N/A
P1-split GAL4	Gift from David Anderson[6]	N/A
$TAR3^{\Delta 29}$	Gift from Edward Blumenthal[46]	N/A
$TAR2\text{-}Tar3^{\Delta 124}$	Gift from Edward Blumenthal[46]	N/A
TAR1	Gift from Carsten Duch[47,48]	N/A
$T\beta h^{nm18}$	Gift from Henrike Scholz and Sarah Certel	N/A
$Tdc2^{RO54}$	Gift from Henrike Scholz	N/A
SPN/IPS-split-GAL4	Gift from Thomas Preat and Pierre-Yves Plaçais[77]	N/A
AstC-GAL4 (II)	Gift by Kim Young-Joon	N/A
AstC-GAL4 (III)	Gift by Kim Young-Joon	N/A
$tub\text{-}GAL80^{TS}$	Bloomington DSC	RRID: BDSC_7017
UAS-Dicer	Bloomington DSC	RRID: BDSC_24650
71G01-GAL4	Gift by David Anderson[6]	N/A
Software and algorithms		
R scripts for Behavioral Analysis	This study	https://github.com/salonirose95/Behavioural-Probability

RESOURCE AVAILABILITY

Lead contact

Further information and requests for resources and reagents should be directed to and will be fulfilled by the Lead Contact Carolina Rezaval (c.rezaval@bham.ac.uk).

Materials availability

This study did not generate new unique reagents.

Data and code availability

- All data reported in this paper will be shared by the lead contact upon request.
- R code used to analyze the behavioral data is available in the public repository at the following link: https://github.com/salonirose95/Behavioural-Probability.
- Any additional information required to reanalyze the data reported in this work paper is available from the Lead Contact upon request.

EXPERIMENTAL MODEL AND SUBJECT DETAILS

All fly lines used in this study are described in the Key resources table. All *Drosophila melanogaster* strains were reared at 25°C and 40%–50% humidity on standard cornmeal-agar food in 12 h light: dark cycle. Canton-S strain flies were used as wild-type.

METHOD DETAILS

Behavioral assays

Male flies were individually raised on standard cornmeal food until they were 5-7 days old and were maintained on a 12 h light-dark cycle. Virgin females used were 3-8 days old and were decapitated immediately before the experiment. Males were individually wet-starved in 1% agar vials with a strip of Whatman filter paper (approx. 3 cm long) in each for 24 hours. 5-7 day old fed males of the same genotype were used as controls for the starvation experiments.

Behavioral Choice Assay

Individual male flies were paired with individual decapitated CS virgin females and food (1% (w/v) agar and 0.4% (w/v) Brilliant Blue FCF dye (obtained from Sigma-Aldrich) in 100 mM sucrose solution in 2 cm wide courtship chambers. Behaving flies were videotaped for 15 min using a Sony DCR-HC38 camera and their initial choice was quantified. For experiments with a mobile female, 5 cm wide chambers were used.

Courtship Preference Index (CPI)

Initial choice of a fly was categorized as courting or feeding if it performed either behavior for at least 5 s. Initial choice was monitored for 10 males per genotype/ per replicate. Courtship preference index (CPI) was calculated for each replicate as: $(n^{\text{court first}} - n^{\text{feed first}})/n^{\text{total}}$. Only flies that touched the food and passed by the female before choosing to either court or feed, were considered for analysis as they have perceived both of the stimulus.

Courtship and feeding assays

Male courtship index

Individual male flies were paired with individual decapitated CS virgin females and the percentage of time in which the male displayed any of the courtship steps (including following, tapping, wing extension, licking, and attempted copulation) was measured for 15 minutes and recorded as the male courtship index.

Feeding index

Individual male flies were paired with food (1% (w/v) agar and 0.4% (w/v) blue dye in 100 mM sucrose solution in courtship chambers. Feeding index was calculated as the percentage of time in which the male spent feeding (extending its proboscis) on or near the food patch for 15 minutes.

The behavior of the flies (percentage of time spent feeding or courting) during the observatory period was quantified manually and behavioral plots (ethograms) were generated on Behavioral Observation Research Interactive Software (BORIS).[77] A customized R-code was written to extract information from the ethograms and plot the behavioral probability over time. For the experiments involving neurogenetic manipulations, 20 flies per genotype were randomly chosen using a random number generator for which the ethograms were created.

Direct and Indirect switch

Twenty-four hour starved wild-type flies that switched to courtship after feeding were described to have made a 'direct' or 'indirect switch' based on their trajectory from the food to the female. Male flies that immediately approached the female in a straight line from the food were described to have 'directly' switched to courtship. Flies that explored their surroundings (moved around the perimeter of the chamber more than once) before reaching the female were classified as 'indirect switch'. Time to courtship initiation as flies stopped feeding was calculated and presented in seconds for both groups of flies.

FlyPAD feeding assays

flies were analyzed in flyPAD feeding arenas as previously described in Itskov et al.[37] Single flies (24 hr starved or fed) were gently aspirated into the arenas with one of the two wells loaded with 100 mM sucrose in 1% agarose and were allowed to feed for 30 min. Behaving flies were filmed simultaneously using a Sony DCR-HC38 camera and flies that did not touch the food during the first 20 minutes of observation were excluded from the analysis. Total number of sips taken by individual flies in 15 min (Figure S1E) and 30 min was calculated in MATLAB using custom-written software as described in Itskov et al.[37]

Thermogenetic neuronal activation

All TrpA1 neuronal activation experiments were carried out at 28-29°C on a heated block. TrpA1 candidate males were raised at 18°C on cornmeal food for 4-6 days and were pre-warmed at 28°C for 10 minutes prior to experimentation and tested for behavioral choice at 28-29°C. Flies of the same genotype were tested at 22°C to control for the high temperature experiments.

TARGET system silencing experiments

Flies containing tub-Gal80^{TS}, UAS-TNT and genetic controls were raised at 18 °C. Post eclosion, adult flies were kept at 31°C to inactivate tub-Gal80^{TS} for 2-5 days before testing them at 28°C. Control adult flies were kept at 18°C and tested at 20-22°C.

qRT-PCR

For quantitative RT-PCR, brains were dissected from 24 h starved or non-starved Canton-S male flies (n = 20) and total RNA was extracted using Total RNA Purification Plus Kit (Norgen Biotek). 1 ng-1 µg RNA was reverse transcribed to cDNA using FastGene Scriptase II cDNA synthesis kit (Nippon Genetics) using oligo dT primers in accordance with the manufacturer's protocol. SensiFAST SYBR Hi-ROX Kit (Bioline) and primers targeting Tβh or tdc2 were used to perform quantitative real-time PCR on ABI PRISM 7000 Sequence Detection System. Each experiment was performed in technical triplicates. Rp15 was used as an internal control for normalization. Table S1 lists the sequence of primers used.

Immunohistochemistry and fluorescence intensity quantification

Flies were reared at 25°C and aged for 4-7 days prior to dissection. Starved male flies were wet-starved in 1% agar for 24 h prior to dissection. Flies were dissected in ice-cold 1x PBS (phosphate buffered saline) and fixed in 4% paraformaldehyde for 20 min at room temperature prior to washing at least 5 times for 15-20 min with PBST (0.3% Triton X-100 in PBS). Samples were incubated with anti-nc82 mouse antibody (1:250) with one of the following antibodies: anti-nc82 (DSHB) (1:500), anti-tdc2 rabbit (Fisher) (1:500), anti-TβH rat (gift from Maria Monastirioti) (1:300), anti-GFP chicken (Abcam) (1:1000) or anti-AstC rabbit (gift from Jan Veenstra) (1:100) in 5%–10% normal goat serum (NGS) for 2 days. Following washing, samples were incubated with AF 543 goat anti-mouse (1:500) and AF 546 goat anti-rabbit (1:500) or AF 633 goat anti-rabbit (1:500) or AF 594 goat anti-rat (1:500) or AF 488 goat anti-chicken (1:1000) in 5%–10% NGS for 1 day. All secondary antibodies were obtained from ThermoFisher Scientific (Waltham, MA). Samples were washed and mounted in VectaShield (Vector Lab) mounting medium prior to imaging using a Leica SP8 confocal microscope and images were processed using Fiji (ImageJ) for quantification and Imaris for 3D image processing. Fiji (ImageJ) version 2.0.0-rc-71/1.52p and Imaris version 9.1 were used for image analysis. For confocal image quantification, z-projections were made from 40x oil objective images and the sum of slices was used to quantify integrated fluorescence density. Average fed integrated fluorescence intensity values for each biological replicate were conducted and all fed and starved values normalized to this average to account for variation between experiments.

Two-photon Calcium Imaging

Virgin males were individually raised for 4-6 days post-eclosion. Single males were either maintained in vials with a strip of Whatman filter paper and food (fed) or 1% agar (starved) for 24 h and kept at 25°C. For TyrR^{GNG} and TyrR^{SMP} imaging, we used UAS-GCaMP6s; TyrR-GAL4 males, for TyrR^{PLP} imaging, we used *UAS-GCaMP6s/61A01-LexA; LexAopGa80/TyR-GAL4* males and *UAS-ASAP2/61A01-LexA; LexAopGa80/TyrR-GAL4* males, for TyrR^{IPS} imaging, we *used SPN-GAL4/UAS-GCaMP6s,* for P1 imaging, we used *71G01-GAL4/UAS-GCaMP6s*, for experiments with the TAR3 RNAi, we either tested *UAS-GCaMP6s/+; UAS-TAR3 RNAi/71G01-GAL4* males or *UAS-GCaMP6s/+;TyR-Gal/UAS-TAR3 RNAi*. After 24 hr, flies were prepared for imaging. In brief, flies were immobilized on ice and mounted in a custom-made chamber allowing free movement of the antennae and legs. The head capsule was opened under room temperature carbonated (95% O2, 5% CO2) buffer solution. For fed males, the following buffer was used: 103 mM NaCl, 3 mM KCl, 5mM N-Tris, 10 mM trehalose, 10 mM glucose, 7mM sucrose, 26 mM NaHCO3, 1mM NaH2PO4, 1.5 mM CaCl2, 4mM MgCl2, osmolarity 275 mOsm, pH 7.3; For starved males, a sugar-free buffer was used: 108 mM NaCl, 5 mM KCl, 5 mM HEPES, 15 mM ribose, 4 mM NaHCO3, 1mM NaH2PO4, 2 mM CaCl2, 8.2 mM MgCl2, osmolarity 272 mOsm,

CellPress
OPEN ACCESS

pH 7.3. The fly, in the recording chamber, was placed under the Two-Photon microscope (Scientifica). Fluorescence was excited using ~140 fs pulses, 80 MHz repetition rate, centered on 910 nm generated by a Ti-Sapphire laser (Chameleon Ultra II, Coherent). mages of 256 X 256 pixels were acquired at 5.92 Hz, controlled by ScanImage 3.8 software.[78]

For tyramine or octopamine experiments, a tyramine or octopamine (Sigma-Aldrich) solution (1 mM or 5 mM) was prepared on the day of the experiments, in either normal carbonated buffer (for fed flies) or sugar-free carbonated buffer (for starved flies). The head capsule of the fly was continuously perfused with the respective carbonated buffer or tyramine solution at about 2 ml/min using a Watson-Marlow 120S/DV WM Sci Q400-1H1D perfusion system. GCaMP signals were recorded immediately before tyramine or octopamine application, during the tyramine application and after 5 min of washout with the buffer. Tyramine or octopamine was applied for 90 s and all recordings were 94 s long. P1 neurons did not respond to 1 mM tyramine (data not shown) but only to 5 mM.

For analysis, two-photon fluorescence images were manually segmented using Fiji.[79] Movement of the animals was small enough such that the images did not require registration. For subsequent quantitative analyzes, custom Fiji and MATLAB scripts were used. The baseline fluorescence, F0, was defined for each stimulus response as the mean fluorescence F from first the 4 s of recording. F/F_0 accordingly describes the fluorescence relative to this baseline. After this 4 s, the mean fluorescence of the GCaMP signal was measured for a period of 90 s.

For sucrose (Sigma-Aldrich) feeding experiments, the flies were prepared as previously, however, to reduce brain movements induced by feeding, a drop of 1% agar in sugar-free carbonated buffer was placed on top of the brain and the rest of the chamber was filled with sugar-free buffer. For the sucrose presentation, a 12 μL drop of 100 mM sucrose in water (with brilliant blue FCF [FUJIFILM Wako] dye: 0.4%) was positioned close to the fly and the GCaMP signal was obtained. The F_0 was established as before, the next 16 s of recording were used as the pre-feeding condition and then the drop of sucrose was positioned in close proximity to the proboscis (the fly was allowed to feed for 60 s) and then the sucrose was removed, and the recording continued for 20 s more. Total recording length 100 s. For the analysis, only flies that clearly showed consumption of the sucrose (blue color could be seen through the translucent abdomen) were used for the quantification. For the pre-feeding condition, the mean fluorescence over 16 s was used; for the sucrose feeding condition, the mean fluorescence over 60 s of feeding was used. ASAP2 experiments follow the same protocol. GCaMP6s data are presented as $\Delta F/F_0$. ASAP2 data are presented as $-\Delta F/F_0$ to correct the inverse relation between sensor fluorescence and membrane voltage.

HPLC

Dissected heads were homogenized in 50 μL of 0.1 M cold perchloric acid, before being centrifuged for 30 min at 30,000 x *g*. HPLC analysis of the supernatant was performed using an Ultimate 3000 UHPLC by ThermoFisher Scientific, equipped with a Kinetex 150 × 2.1 mm C_{18} column. The chromatographic conditions are as follows: 10 μL injection, column temperature at 25°C, 0.2 ml·min^{-1} flow rate, fluorescence detection (λex/λem = 274/304 nm). Separation was achieved using a linear gradient of 20 mM potassium phosphate buffer, pH 3.5 to 80% acetonitrile over 40 min and tyramine standards were used for peak identification and quantitation. Data are from four separate experiments and represented as the ratio of tyramine detected from 40 starved and 40 fed flies.

QUANTIFICATION AND STATISTICAL ANALYSIS

All statistical analysis was performed using the GraphPad Prism software v8 and R (version 3.6.3). Shapiro-Wilk normality test was used to assess whether the data followed a normal distribution. P values were calculated using either Mann-Whitney (non-parametric Student's t test) or Kruskal-Wallis tests followed by Dunn's multiple comparison test. For calculation of cliff's delta, R package 'effsize' was used. Ca^{2+} imaging data were compared by a paired t test for normally distributed data, otherwise a Wilcoxon matched-pairs signed rank test was used for non-Gaussian distributed data. Each n corresponds to a recording from a single fly.

Current Biology, Volume *31*

Supplemental Information

A neuronal mechanism controlling the choice between feeding and sexual behaviors in *Drosophila*

Sherry J. Cheriyamkunnel, Saloni Rose, Pedro F. Jacob, Lauren A. Blackburn, Shaleen Glasgow, Jacob Moorse, Mike Winstanley, Patrick J. Moynihan, Scott Waddell, and Carolina Rezaval

Supplementary information

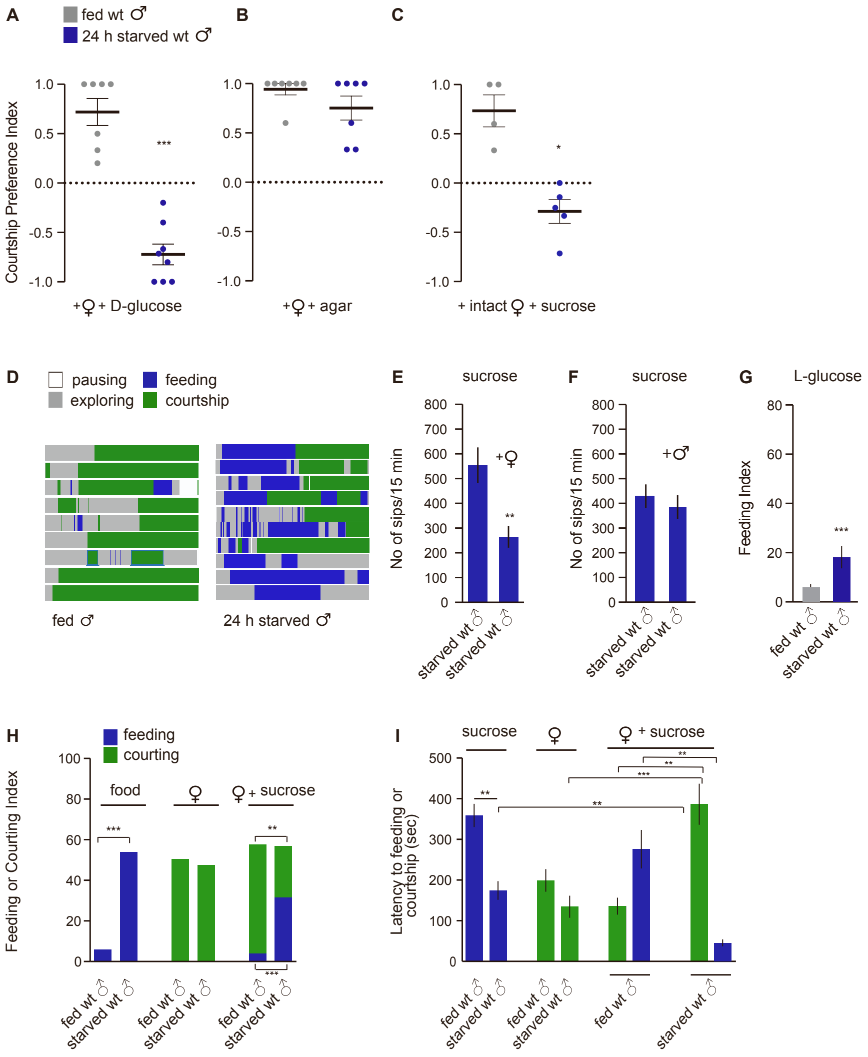

Figure S1. Analysis of feeding and courtship dynamics in sated and 24 h starved male flies, related to Figure 1.

(A-B) Courtship Preference Indices obtained from groups of fed and 24 h starved males choosing between courting a female or feeding on agar with D-glucose (A) (n=36-68 flies) or plain agar (B) (n=29-35 flies).

(C) Courtship Preference Indices obtained from groups of fed and 24 h starved males choosing between courting an intact, mobile female or feeding on sucrose (n=18-29).

(D) Representative ethograms of fed and starved Canton S males during 15 min in the presence of sucrose and a female (n=10).

(E) Quantitative feeding of flies on sucrose with or without a female, measured as number of sips in 15 min using flyPAD (16-22).

(F) Quantitative feeding of flies on sucrose with or without a decapitated male, measured as the number of sips in 15 min using flyPAD (n=44-58).

(G) Percentage of time spent by fed or 24 h starved CS males feeding on L-glucose (n=26-29).

(H) Percentage of time spent feeding on sucrose or courting the female during the observation period, in the presence of either stimulus or both (n=29-33).

(I) Latency in seconds (s) to the preferred action (feeding or courtship) of 24 h starved or fed males (n=26-30).

All bar graphs are shown as mean ± SEM, whereas the mean percentage time spent feeding or courting is shown in (H). Scatter plots show Courtship Preference Indices obtained from different groups of flies treated as independent biological replicates. Lines show the mean and error bars indicate SEM. *p < 0.05, **p < 0.01, ***p<0.001 calculated using Mann-Whitney U tests for in (A, B, C, E, F and G) and Kruskal Wallis test followed by Dunn's post-hoc multiple comparison test performed in (H, I). Absence of * denotes non-significant data.

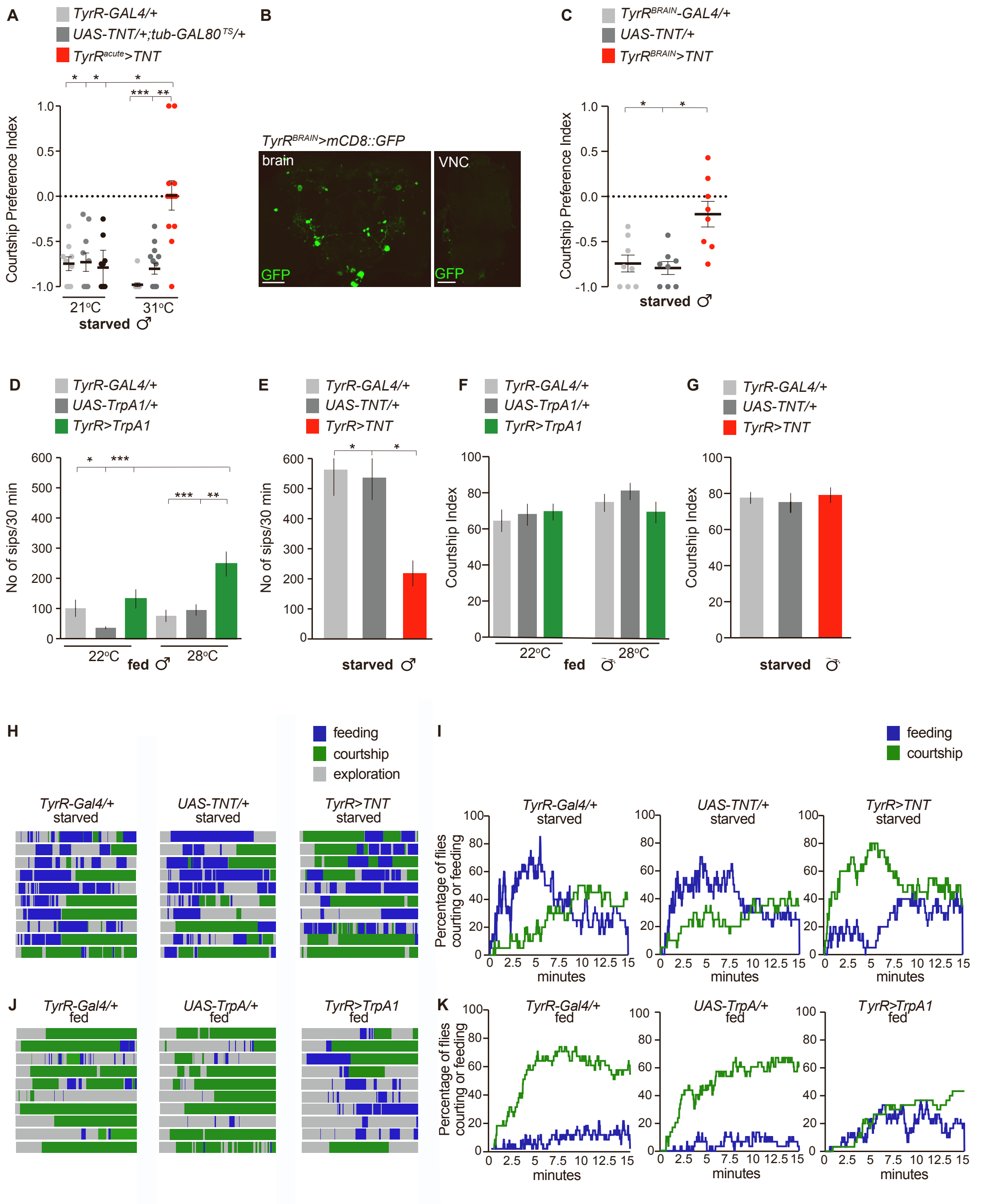

Figure S2. Behavioral effects of manipulating TyrR and TyrR^{IPS} neurons in starved or fed male flies, related to Figure 2.

(A) Acute silencing of TyrR neurons alters courtship preference. Courtship Preference Index of *UAS-TNT/+; tub-GAL80^{TS}/TyrR-GAL4* (*TyrR^{acute}>TNT*) males and genetic controls tested at 21°C and 31°C (n= 56-89).

(B) *UAS-mCD8-GFP* expressed under the control of *TyrR^{BRAIN-}GAL4* in the adult brain and ventral nerve cord. (Genotype used: *Otd-FLP/UAS>stop>mCD8::GFP;TyrR-GAL4/+).*

(C) Courtship Preference Index of *Otd-FLP/UAS>stop>TNT; TyrRGal4/+* (*TyrR*^{brain}*>TNT*) males and genetic controls (n = 52-60).

(D) Food intake of fed *TyrR-GAL4/UAS-TrpA1* and genetic control males paired with 100 mM sucrose measured as sips taken in 30 min in a flyPAD setting, tested at the control (22°C) and activation (28°C) temperatures, respectively (n=34-73).

(E) Food intake as number of sips taken during 30 min in 24 h starved *TyrR-GAL4/UAS-TNT* and genetic control males using flyPAD (n=26-40).

(F) Time spent courting in 15 min of fed *TyrR-GAL4/UAS-TrpA1* males and controls (n=16-30).

(G) Time spent courting in 15 min of starved *TyrR-GAL4/UAS-TNT* males and controls (n=23-31).

(H) Representative ethograms of *TyrR-GAL4/UAS-TNT* males and their respective genetic controls during 15 min in the presence of sucrose and a female (n=20).

(I) Percentage of *TyrR-GAL4/UAS-TNT* males (and their genetic controls) displaying either courtship or feeding behavior during 15 min in the presence of sucrose and a female in 1 s time bins (n=20).

(H) Representative ethograms of *TyrR-GAL4/UAS-TrpA* males and their respective genetic controls during 15 min in the presence of sucrose and a female (n=20).

(I) Percentage of *TyrR-GAL4/UAS-TrpA* males (and their genetic controls) displaying either courtship or feeding behavior during 15 min in the presence of sucrose and a female in 1 s time bins (n=20).

Scatter plots show Courtship Preference Indices obtained from different groups of flies treated as independent biological replicates. Lines show the mean and error bars indicate SEM. *p < 0.05, **p < 0.01, ***p<0.001 calculated using Kruskal Wallis followed by Dunn's post-hoc multiple comparison test in A, C, D, E, F and G. In A, D and F the means obtained from the experimental groups were compared to both genetic controls tested at the control and activation temperatures and to data from the same genotype at the control temperature.

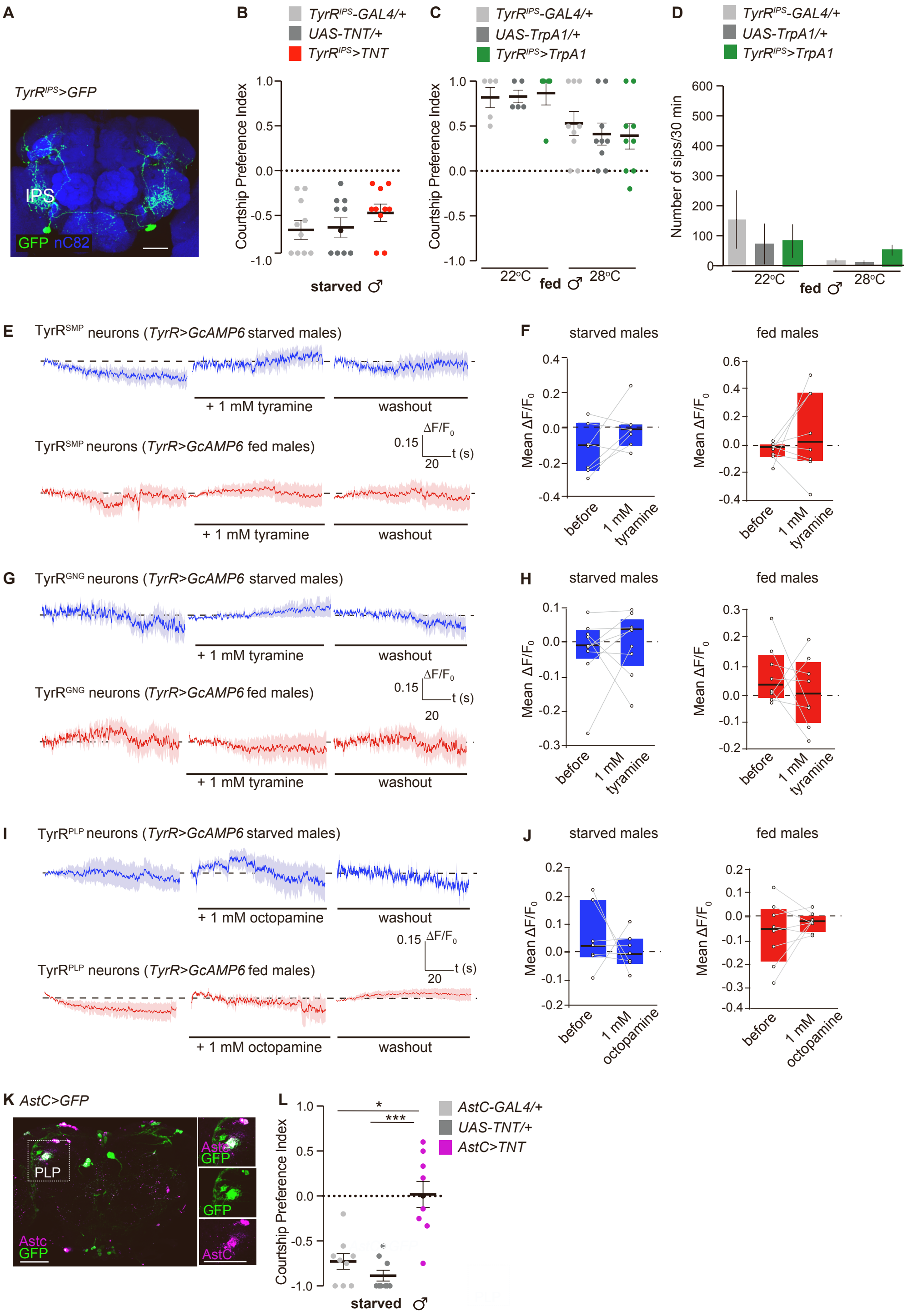

Figure S3. TyrR^{SMP}, TyrR^{GNG} and TyrR^{PLP} neurons response to tyramine and octopamine in starved and fed males, related to Figure 4.

(A) *UAS-mCD8-GFP* expressed under the control of IPS split-GAL4 in the adult brain. GFP staining (green); neuropil counterstained with anti-nC82 (blue). Scale bar= 50 µm.

(B) Courtship Preference Indices obtained from 24 h starved *IPS split-GAL4/UAS-TNT males*, choosing between feeding on sucrose and courting a female (n=57-64).

(C) Courtship Preference Indices of fed *IPS split-GAL4/UAS-TrpA1* and genetic control males, allowed to choose between feeding on sucrose and courting a female, at the control (22°C) and activation (28°C) temperatures (n= 40-60).

(D) Food intake of fed *IPS split-GAL4/UAS-TrpA1* and genetic control males paired with 100 mM sucrose measured as sips taken in 30 min in a flyPAD setting, at the control (22°C) and activation (28°C) temperatures (n=4-39).

(E) Application of 1 mM of tyramine (Tyr; middle panel) did not change the GCaMP6s Ca^{2+} response of $TyrR^{SMP}$ neurons in either starved (blue) or fed (red) males, when compared to the response before application (left panel). The $\Delta F/F_0$ represents the evoked fluorescence change from baseline. Traces were averaged from 8 flies. Solid line represents mean and shaded areas indicate SEM. Genotype tested: *UAS-GCaMP6s; TyrR-GAL4*.

(F) Quantification of the mean $\Delta F/F_0$ responses of $TyrR^{SMP}$ neurons using GCaMP6s over 90 s before (pre) and after the application of Tyr (1 mM Tyr) in starved (blue) and fed (red) males. No changes to 1 mM Tyr were observed in starved (n=8) or fed males (n=8).

(G) Application of 1 mM of tyramine (Tyr; middle panel) did not change the GCaMP6s Ca^{2+} response of $TyrR^{GNG}$ neurons in starved (blue) or fed (red) males, in comparison to the response before application (left panel). Traces were averaged from 8-9 flies. Genotype tested: *UAS-GCaMP6s; TyrR-GAL4*.

(H) Quantification of the mean $\Delta F/F_0$ responses of $TyrR^{GNG}$ neurons using GCaMP6s over 90 s before (pre) and after the application of Tyr (1 mM Tyr) in starved (blue) and fed (red) males. No changes to 1 mM Tyr were observed in starved (n=9) or fed males (n=8).

(I) Application of 1 mM of octopamine (middle panel) did not change the GCAMP6s Ca^{2+} response of $TyrR^{PLP}$ neurons in both starved (blue) and fed (red) males, in comparison to the response before application (left panel). Traces were averaged from 7-8 flies. Genotype tested: *UAS-GCaMP6s/61A01-LexA; LexAopGa80/TyrR-GAL4.*

(J) Quantification of the mean $\Delta F/F_0$ responses of TyrR^{PLP} neurons (measured by GcAMP6s) over 90 s before (pre) and after the application of Oct (1 mM Oct) in starved (blue) and fed (red) males. No changes to 1 mM Oct were observed in starved (n=7) or fed males (n=8).

(K) UAS-mCD8-GFP expressed with AstC-GAL4 (III) in the adult brain. Anti-GFP (green) and anti-AstC (magenta) staining is shown. Scale bars= 50 μm or 25 um, insets.

(L) Courtship Preference Indices of 24 h starved *AstC-GAL4(III)/UAS-TNT* and genetic controls (n=50-61).

Lines show the mean and error bars indicate SEM. All boxplots represent 25th to 75th percentile and black line as the mean. *p < 0.05, **p < 0.01, ***p<0.001 calculated using paired t-test in (F, H and J for fed), Wilcoxon matched-pairs signed rank test in (J for starved) and Kruskal Wallis test followed by Dunn's post-hoc multiple comparison in (B, C, D and L). Absence of * denotes non-significant data.

Table S1: List of primers used for qRT-PCR in this study, Related to STAR Methods.

Primer	Sequence
Rp15 forward	AGG ATG CAC TTA TGG CAA GC
Rp15 reverse	GCG CAA TCC AAT ACG AGT TC
Tβh forward	ACA ATG TAC GTG GTT TGG GC
Tβh reverse	AGT ATC TTG TCG GCC CGT AG
Tdc2 forward	ACG CAT TGG CAG CAT CCT C
Tdc2 reverse	TGG CAG CAA GCA TCG TGA C
